# Effectiveness of acupuncture for angina pectoris: a systematic review of randomized controlled trials

**DOI:** 10.1186/s12906-015-0586-7

**Published:** 2015-03-28

**Authors:** Changhe Yu, Kangshou Ji, Huijuan Cao, Ying Wang, Hwang Hye Jin, Zhe Zhang, Guanlin Yang

**Affiliations:** First Clinical Department, Liaoning University of Traditional Chinese Medicine, Shenyang, Liaoning China; Center for Evidence-Based Chinese Medicine, Beijing University of Chinese Medicine, Beijing, China; Center of Education and Laboratory, Liaoning University of Traditional Chinese Medicine, Shenyang, Liaoning China; Oriental medical college, Kyung Hee University, Dongdaemun-gu, Korea; Affiliated Hospital of Liaoning University of Traditional Chinese Medicine, Shenyang, Liaoning China; Liaoning University of Traditional Chinese Medicine, Shenyang, Liaoning China

**Keywords:** Acupuncture, Angina pectoris, Systematic review, Randomized controlled trial, Meta-analysis

## Abstract

**Background:**

The purpose of this systematic review is to assess the effectiveness of acupuncture for angina pectoris.

**Methods:**

Eleven electronic databases were searched until January 2013. The study included randomized controlled trials that the effectiveness of acupuncture alone was compared to anti-angina medicines (in addition to conventional treatment) and the effectiveness of a combination of acupuncture plus anti-angina medicines was compared to anti-angina medicines alone. The trial selection, data extraction, quality assessment and data analytic procedures outlined in the 2011 Cochrane Handbook were involved.

**Results:**

The study included 25 randomized controlled trials (involving 2,058 patients) that met our inclusion criteria. The pooled results showed that the number of patients with ineffectiveness of angina relief was less in the combined acupuncture-anti-angina treatment group than in the anti-angina medicines alone group (RR 0.33, 95% CI 0.23-0.47, p < 0.00001, I2 = 0%). Similarly, compared to the anti-angina medicines alone group, fewer patients in the combined treatment group showed no ECG improvement (RR 0.50, 95% CI 0.40-0.62, p < 0.00001, I2 = 0%). However, no differences were observed between acupuncture treatment alone and anti-angina medicines alone for both outcome measures. Only four trials mentioned adverse effects. One trial found no significant difference between acupuncture and Chinese medicine, and three reported no adverse events. The quality of the trials was found to be low.

**Conclusions:**

The findings showed very low evidence to support the use of acupuncture for improving angina symptoms and ECG of angina patients. However, the quality of the trials included in this study was low. Large and rigorously designed trials are needed to confirm the potential benefit and adverse events of acupuncture.

**Electronic supplementary material:**

The online version of this article (doi:10.1186/s12906-015-0586-7) contains supplementary material, which is available to authorized users.

## Background

Angina pectoris (AP) is the result of myocardial ischemia, a clinical condition typified by the symptoms of intense tightness or heavy pressure in the chest, radiating to the neck, jaw, shoulder, back, arm and epigastric region [1]. Angina, consisting of stable and unstable angina, is a common symptom of coronary artery atherosclerotic disease [2]. AP is also a sign that someone is at an increasing risk of heart attack, cardiac arrest, and sudden cardiac death [3].

The World Health Organization (WHO) report [4] on the global burden of disease indicated that ischemic heart disease was the number one cause of death (out of a total of 136) in the world, accounting for 12.2% of all deaths. This figure is predicted to rise to 14.2% by 2030. In the USA and the UK, the incidence of AP was reported as 6 to 30 per 1000 men, and 4 to 16 per 1,000 women [5,6]. In China, the incidence of coronary artery disease increased by 26.1% for males and 19.0% for females from 1998 to 2008 [7].

The American College of Cardiology and American Heart Association (ACC/AHA) guidelines [3,8] recommend anti-ischemic therapies and/or anti-platelet/anti-coagulation therapies for unstable angina and to control necessary risk factors for stable angina. However, these therapies all have risks: the nitrates used for anti-ischemic treatment may cause drug resistance; nitroglycerin may be of no benefit to survival; anti-platelet/anti-coagulation may cause intracranial bleeding and gastrointestinal haemorrhage; beta blockers may cause physical and mental fatigue, heart failure, heart blockages, and bronchospasms; calcium antagonists may cause headache, flushing, dizziness and bradycardia, atrium ventricle (AV) dissociation, and AV block; angiotensin converting enzyme inhibitors (ACEI) may cause hyperkalemia and cough; statins could possibly cause other harm with myopathy and hepatotoxicity [9,10]. Revascularization may also lead to complications [11].

In China, Chinese patent medicines, such as Suxiao Jiuxin Wan, Di’ao Xinxuekang, and Danshen agents are popular treatments for AP [12-18]. All of these appear to be effective in the treatment of angina pectoris and no serious side effects have been identified [12,13]. Specifically, Compound Danshen pills are more effective than Di’ao Xinxuekang and isosorbide dinitrate [13-17]. However, Suxiao Jiuxin Wan can lead to gastrointestinal reactions in some cases, but these can be relieved by taking the medication after a meal [18].

The evidence for acupuncture as an effective treatment of cardiovascular risk factors for chronic stable angina was considered in the ACC/AHA guidelines published in 1999. The evidence was not considered supportive for an effective treatment of acupuncture for this indication [19]. However, acupuncture has been suggested as a complementary and alternative treatment to assist in the treatment of cardiovascular risk factors for angina such as hypertension [20], smoking cessation [21], hyperlipidemia [22], and weight control [23,24]. In China, acupuncture is usually regarded as a major therapy for stable or unstable angina pectoris in addition to the conventional treatments such as taking nitroglycerin. This is due to the shortage of medical resources [25] and the lower cost of acupuncture treatments [26] compared with medicines and revascularizations. Still, acupuncture could be used for treating other cardiovascular diseases, such as heart failure [27].

According to the Traditional Chinese medical theory, the mechanism of acupuncture therapy may be that of regulating qi, blood, yin, and yang to reinforce health and eliminate pathogenic factors [28]. Acupuncture can also normalize the cardiac nervous system [29], activate the electro-magnetic signals and the opioid systems [30], dilate coronary arteries and reduce oxygen demand [31], stimulate the somato-autonomic nerve reflex [32], and possibly attenuate the sympathoexcitatory cardiovascular reflex response [33].

Many trials have tested the usefulness of acupuncture for angina; however, its effectiveness at relieving symptoms and improving the functional capacity of patients with angina pectoris has been inconsistent [34-36]. Therefore, the true value of this technique has yet to be determined. The objective of this systematic review was to assess the effectiveness of acupuncture for angina pectoris based on randomized controlled trials (RCTs).

## Methods

### Eligibility criteria

Trial design: parallel randomized controlled trials (RCTs) regardless of blinding method. If randomized cross-over trials were collected, first phrase treatments were included. We included published and unpublished trials. No restrictions were applied regarding the language of publication. Multiple publications reporting the same comparisons of participants were combined into one study.

Participants: patients with angina pectoris, with diagnoses according to national or international criteria, regardless of gender, age, race, educational or economic status, were included.

Interventions and comparisons: the trials employing acupuncture therapy (manual acupuncture, electro-acupuncture and auricular acupuncture) versus sham acupuncture (non-penetrative and penetrative sham at non-acupuncture points), usual care, invasive treatments, anti-angina medicines (effective Chinese patent medicines in this case) or no intervention (waiting list) were included. Combination therapies of acupuncture and non-acupuncture interventions versus non-acupuncture elements were also included. The paper attempted to analyze the two cases, that is, to compare the effectiveness of acupuncture and non-acupuncture treatment as well as the effectiveness of acupuncture plus non-acupuncture and non-acupuncture.

Excluded interventions were the use of acupressure, the tip of a bone or bamboo or wood, laser, moxibustion, drugs injection, catgut implantation, plaster or ultrasonic stimulation of acupoints, or spinal cord stimulation, or needle bleeding for the treatment. Combinations of moxibustion and acupuncture or the combination of herb decoction and acupuncture were excluded as well.

Primary outcomes for effectiveness included i. overall mortality, ii. cardiovascular mortality, iii. cardiovascular events including Acute Myocardial Infarction (AMI), Percutaneous Transluminal Coronary Angioplasty (PTCA) or Coronary Artery Bypass Grafting (CABG), and iv. functional outcomes (e.g. number of angina episodes, severity of angina, clinical comprehensive outcome measures, shortness of breath returned to normal).

Secondary outcomes included i. health-related quality of life, ii. electrocardiograph (ECG), iii. dynamic electrocardiogram (DCG), iv. consumption of existing anti-angina drugs, v. re-admission to hospital, vi. psychosocial function outcomes (such as the Hamilton depression scale), vii. ejection fraction (EF) and vii. adverse event outcomes. All the effective outcomes were measured after treatments regardless of the exact time point, but after at least 10 days or one course defined by the trials. All of the adverse event outcomes were reported at any time during the trials. Adverse event outcome included 1) mortality, 2) life-threatening events (e.g. acute laryngeal edema), 3) any event that results in the discontinuation of treatment (e.g. jeopardization of the patient or required intervention to prevent it), 4) persistent or significant disability, and 5) acupuncture-related unexpected events (e.g. subcutaneous bleeding, pain complaints).

### Search strategy

A comprehensive and exhaustive search strategy was formulated in an attempt to identify all relevant trials. Eleven electronic databases were searched: Cochrane Central Register of Controlled Trials (CENTRAL, to 2013), PUBMED (1966 to 2013), ExcerptaMedica Database (EMBASE and EMBASE CLASSIC, 1974 to 2013), China National Knowledge Infrastructure (CNKI, 1979 to 2013), Chinese biomedical database (CBM, 1978 to 2013), Chinese Scientific journal database (VIP, 1989 to 2013), Wan Fang database (1985 to 2013), Korean Medical Database (the National Digital Science Links [NDSL 1960–2013], the Korea Institute of Science and Technology Information [KISTI 1960–2013], Korean Studies Information Service System [KISS 1996–2013]) and the Japanese Medical Abstracts Society (ICHUSHI before1982-1982-2013). Two reviews available online “Acupuncture: Review and Analysis of Reports on Controlled Clinical Trials” (http://apps.who.int/medicinedocs/pdf/s4926e/s4926e.pdf) and “Current Bibliographies in Medicine Acupuncture Usage Cardiovascular System” (http://www.healthy.net/Health/Article/Current_Bibliographies_in_Medicine_Acupuncture_Usage_Cardiovascular_System/2473) were reviewed for potential eligible trials.

The ongoing trials, whose results have not been published yet, were searched in the following databases: the National Research Register, Clinical Trials, Chinese Clinical Trial Register, the ISRCTN Register, and WHO ICTRP Search Portal. All the searches ended at January 2013. Search text terms and MeSH terms included angina pectoris, angina*, angor*, chest near pain*; acupuncture therapy, acupunctur*, electroacupunctur*, auricular acupuncture*, acupoint*, trigger point*, acupuncture points and acupoints were used and adapted for different databases as necessary. Reference lists from the included trials were searched to find other potential papers (See Additional file 1).

### Data extraction and quality assessment

Two reviewers (Yu CH and Ji KS) independently assessed the studies for inclusion and abstracted the data based on details. Another two reviewers (Wang Y and Jin HH) helped to review and translate Japanese or Korean articles into Chinese or English. Differences in the process of data extraction were resolved through discussions or, if necessary, by consulting the third reviewer (Yang GL). In the case of unclear or missing data, the publication authors were contacted. In cases of author non-response, the reviewers extracted information available in publications. If the number randomized and the numbers analyzed were inconsistent or the reasons for unclear/missing data were unavailable, we reported both numbers of patients in the table in Result section. The reviewers recorded the number of participants experiencing the event in each group of the trial.

Quality of the included trials was evaluated according to the Cochrane Reviewers’ Handbook 2011 [37]. The reviewers assessed trials according to the risk of bias for each important outcome within included trials, including sequence generation, allocation concealment, blinding of participants and personnel, blinding of outcome assessment, incomplete outcome data, selective outcome reporting and other biases, which included the trials’ early end due to the benefits and the baseline imbalance. In the case of unclear information, the publication authors were contacted. In cases of author non-response, the reviewers evaluated trials based on information available in the publication. If the number randomized and the number analyzed were inconsistent and the outcomes mentioned in the “method” and reported in the “results” were inconsistent, the reviewers identified these as inconsistent and having a “high risk of bias.” The quality of all the included trials was categorized as “low risk”, “high risk” or “unclear risk.” Trials were broadly subdivided into the following three categories: Low risk of bias for all key domains: low risk of bias; Unclear risk of bias for one or more key domains: unclear risk of bias; High risk of bias for one or more key domains: high risk of bias. In addition, the Standards for Reporting Interventions in Controlled Trials of Acupuncture (STRICTA) [38] was used to evaluate the reporting quality, including acupuncture rationale, needling details, treatment regimen, co-interventions, practitioner background and control interventions.

### Data analysis

Data were summarized using relative risk (RR) with 95% confidence intervals (CI) for binary outcomes or mean difference (MD) with 95% CI for continuous outcomes. All other outcomes were transformed to binary or continuous outcomes for data analysis. Revman5.1.0 software was used for data analysis. Clinically, various types of acupuncture and different acupoints were included in the acupuncture treatment. In this sense, the random-effect model was chosen for the meta-analysis. If a meta-analysis was possible, the Chi square statistic with significance being set at p < 0.1 and I^2^ statistic would be used to estimate total variation across trials that was due to heterogeneity in percentage, 0% to 40%: might not be important; 30% to 60%: might represent moderate heterogeneity; 50% to 90%: might represent substantial heterogeneity; 75% to 100%: considerable heterogeneity [37].

Possible sources of heterogeneity were assessed by subgroup analyses [37]. Due to the different degrees of severity, acupuncture treatment may have different effects on alleviating the angina. When sufficient data were available, trials were categorized into a stable angina (SAP) group, unstable angina (UAP) group, and coronary heart disease angina pectoris (CHD-AP, which contained a mixture of stable and unstable angina patients) group in order to explore the heterogeneity if it existed. Differences among SAP, UAP, or any kind of angina (CHD-AP) treated by acupuncture also needed verification. In addition, the acupuncture treatment duration for angina was explored through another subgroup analysis based on different course durations, treatment durations, and outcome time points. According to the acupuncture course research [39], 7 to10 days as one course duration and 2 to 3 courses as one treatment were popular and common in the acupuncture treatment, but the changes in courses or treatments depended on the acupuncturists’ experiences and patients’ conditions. Thus, based on the clinical experts’ suggestion, the analyses were done by defining three groups: a short term group of was 10 to 19 days; a moderate term group of 20 to 30 days; and a long term group of more than 30 days. A third subgroup analysis was also conducted for the different types of acupuncture treatments to detect whether different effects existed among electro-acupuncture, needle-embedding, and body acupuncture.

The robustness of results were checked by sensitivity analyses as follows: repeating the analysis excluding unpublished trials, trials with sample size less than 40, and trials with unclear randomization procedure [37].

Publication bias was explored using funnel plot analysis.

Quality of evidence was assessed across important outcomes and the main findings were summarized using GRADE approach to support management recommendations by the software GRADEprofiler (version 3.6). As per GRADE, the researchers further assessed the data with regard to risk of bias, inconsistency, indirectness, imprecision and publication bias.

## Results

### Description of trials

**Figure 1:**
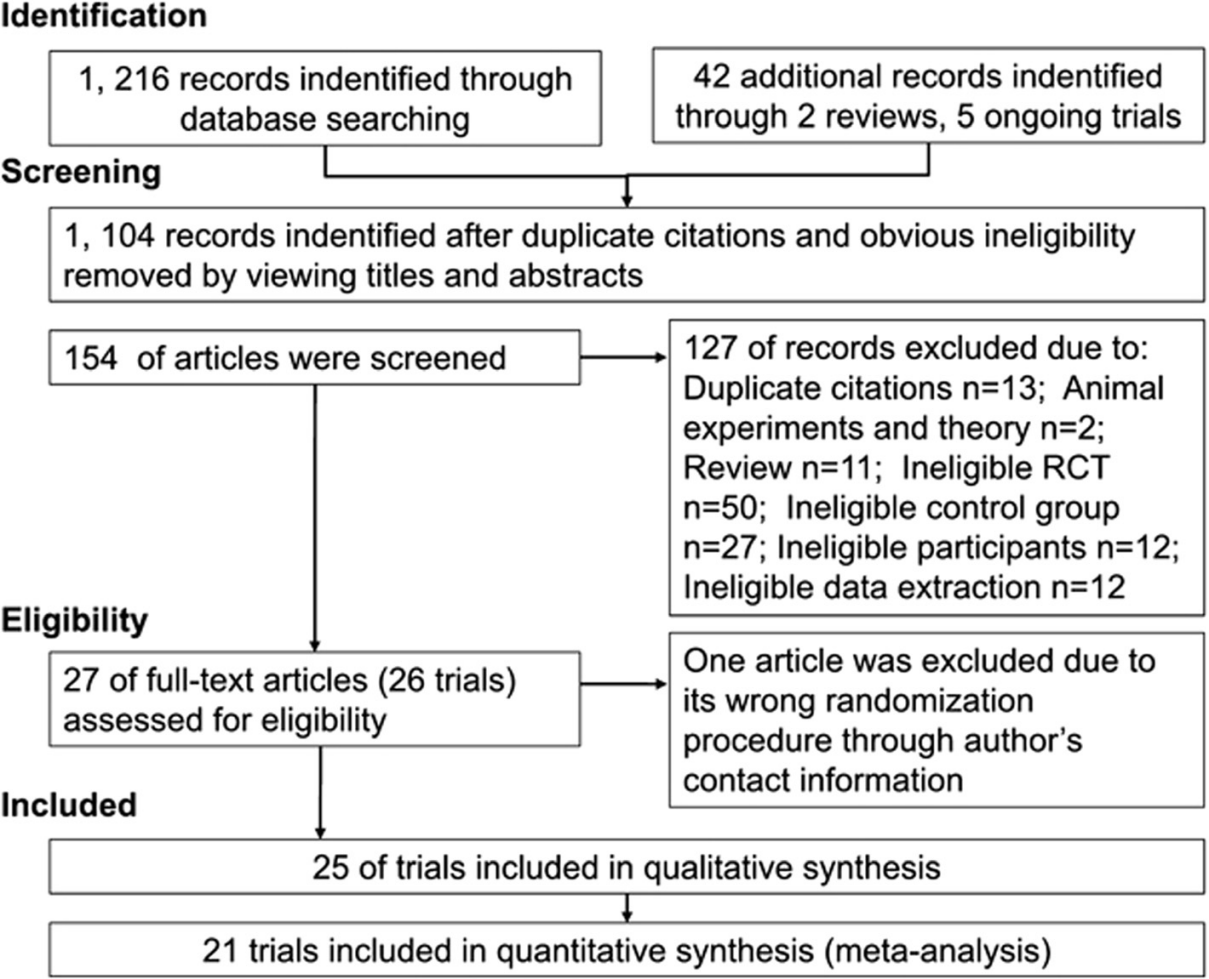
**The process of study selection.**

Primary searches of 11 electronic literature databases, five ongoing trial databases, and two reviews identified 1,258 citations. The majority of these were excluded for obvious ineligibility including irrelevant titles and abstracts (some papers were found in more than one database). A total of 154 full text papers were retrieved. A total of 127 articles were excluded from this review due to duplicate publications; animal experiments and theoretical study; review; non-RCT design; ineligible control groups; ineligible participants; and unavailable data. Due to insufficient information in the reports, twenty-three authors were contacted following Wu’s outline for telephone interviews [40], but only 9 responded and provided us with further information about the methodological evaluation. However, after confirmation from these 9 authors, one of the trials was excluded because of its non-randomization or semi-randomization and no allocation concealment. Thus, 26 articles [35,36,41-64] with 25 trials (23 RCTs and 2 crossover trials [35,36]) were included in this review (Figure 1); and among these were 4 trials [43,46,55,64] including 8 comparisons due to three arms in trials and one trial published in 2 articles [60,61] with different outcomes, while 4 trials [46,49,59,62] were graduate student dissertations. All included trials were evaluated in the qualitative synthesis and 21 trials were included in the quantitative synthesis (meta-analysis). The other four trials [35,36,54,59] were not suitable for meta-analysis due to insufficient data (Study 35 and 36 reported in section 3,3,3; Study 59 reported in section 3.3.1.1) or considerable heterogeneity (Study 54 reported in section 3.3.2.5). The characteristics of included trials are listed in Tables 1 and 2.

**Table 1:** **Summary of included trials basic information**

**Study ID**	**Settings**	**Recruit Sources**	**TG (M/F)**	**CG (M/F)**	**Age (mean or range)**	**Disease history (mean or range)**	**Diagnostic criteria (angina type)**	**AE**
BALLEGAARD 1986 [35]	Denmark	NR	12/1	11/2	TG: 54; CG: 58	NR	Modified WHO/ISFC (SAP)	NO
BALLEGAARD 1990 [36]	Denmark	News	19/5	19/6	TG: 67; CG: 66	NR	Modified WHO/ISFC (SAP)	NO
CAO JP 2002 [41]	MC	In	7/23	5/16	TG: 63; CG: 63	TG: 55.14 m; CG: 61.62 m	WHO/ISFC, IM 4^th^ (UAP)	NR
CHANG PF 2005 [42]	MC	Out	19/11	17/5	TG: 59; CG: 61	TG:6.4y; CG:7.2y	WHO/ISFC, PIM (SAP)	NR
DIAO LH 2006 [43]	MC	NR	8/22	6/24	TG: 57; CG: 57	NR	WHO/ISFC, GCC, PIM (SAP)	NR
	MC	NR	9/21	6/24	TG: 57; CG: 57	NR		NR
HU NK 1997 [44]	MC	In	60	60	59	NR	WHO/ISFC (CHD-AP)	NR
HUANG J 2004 [45]	MC	Out	22/18	21/19	TG:56; CG:57	TG: 5.3y; CG: 5.2y	WHO/ISFC (SAP)	NO
HUANG J2 2004 [46]	MC	Out/In	11/9	7/13	TG: 60; CG: 59	NR	WHO/ISFC, PIM (SAP)	NR
	MC	Out/In	7/13	7/13	TG: 60; CG: 59	NR		NR
LI CP 2005 [47]	MC	Out/In	36	34	64	3.82y	WHO/ISFC (CHD-AP)	NR
LI HJ 2003 [48]	MC	NR	19/17	16/13	TG: 57; CG: 55	TG: 8y; CG: 7.5y	WHO/ISFC (CHD-AP)	NR
LIU JL 2007 [49]	MC	In	20/10	21/9	TG: 57; CG: 57	TG: 9.2y; CG: 8.5y	GCC (UAP)	NR
LIU JR 2010 [50]	MC	Out/In	18/12	19/13	TG: 49; CG: 49	TG: 6.6y; CG: 6.5y	GCC (CHD-AP)	NR
LIU WP 2004 [51]	MC	In	26/6	25/5	TG: 58; CG: 57	TG: 24.8 m CG: 24.5 m	WHO/ISFC (UAP)	NR
LIU WP 2003 [52]	MC	Out/In	19/13	19/12	TG: 58; CG: NR	TG: 24.8 m; CG: 20.6 m	WHO/ISFC (SAP)	NR
LIU YF 2012 [53]	MC	Out/In	18/15	17/16	TG: 66; CG: 65	6 m-15y	2007ACC/AHA (SAP)	NR
TONG YH 2005 [54]	MC	In	42/38	37/23	TG: 61; CG: 60	TG: 2 m-3.5y; CG: 3 m-3y	WHO/ISFC (CHD-AP)	NR
WANG PJ 2011 [55]	MC	In	30	30	53	2-10y	Modified GCC (SAP)	NR
	MC	In	30	30	53	2-10y		NR
WANG X 2000 [56]	MC	NR	18/10	14/7	TG: 57; CG: 53	NR	WHO/ISFC (UAP)	NR
WU CY 2009 [57]	MC	Out/In	39/29	40/28	TG: 48; CG: 48	TG: 26.4 m; CG: 25.6 m	WHO/ISFC (CHD-AP)	NR
WU HH 2005 [58]	MC	In	23/17	19/21	NR	NR	WHO/ISFC (CHD-AP)	NR
XIE ZQ 2003 [59]	MC	In	15/16	26/10	TG: 61; CG: 60	NR	Modified GCC (UAP)	NR
XU GD 2006 [60], TONG YH1 2005 [61]	MC	In	68/52	47/33	TG: 63; CG: 63	TG: 23 m; CG: 2.4 m	WHO/ISFC (CHD-AP)	NR
YU W 2006 [62]	MC	In	17/13	15/15	TG: 67; CG: 66	TG: 8.35y; CG: 8.71y	WHO/ISFC (CHD-AP)	NO
ZHANG L 2011 [63]	MC	NR	19/16	17/13	TG: 62; CG: 61	TG: 6.70y; CG: 6.52y	WHO/ISFC (CHD-AP)	NR
ZHANG LJ 2005 [64]	MC	Out/In	18/22	25/15	TG: 43–70; CG: 43-69	TG: 5 m-20y; CG:2 m-19y.	WHO/ISFC, GCC (CHD-AP)	NR
	MC	Out/In	21/19	25/15	TG: 45–72; CG: 43-69	TG: 6 m-24y;CG: 2 m-19y.		NR

TG, Treatment Group; CG, Control Group; d, day; m, month; y, year; AE, Adverse Event; Out, outpatient; In, inpatient; News, newspaper; NR, No Report; WHO/ISFC, Nomenclature and criteria for diagnosis of ischemic heart disease; IM X^th^, Internal Medicine book (X^th^ version); PIM, Practice of Internal Medicine book; GCC, Government criteria in China (Guidelines for clinical research of traditional Chinese medicine and new medicine and the other 3 National Conferences’ Advice); CHD, coronary heart disease; AP, angina pectoris; SAP, stable angina pectoris; UAP, unstable angina pectoris; MC, Mainland China.

**Table 2:** **Summary of included trials comparisons and outcome measures**

**Study ID**	**Acupuncture Group (no. of points used)**	**Control Group**	**Treat Duration (days)**	**Outcome measures**	**Outcome Type (Primary/secondary; Subjective/objective)**
BALLEGAARD 1986 [35]	(A) BA (3 × 2), 20 min/d, 7d as ACOT; plus none	(B) Sham acupuncture treatment	21, 3 w follow up	Angina attack rate, NTG consumption and general well-being on an ordinal scale	Pr4/Se1, 4; Su/Ob
BALLEGAARD 1990 [36]	(A) BA (3 × 2), 20 min/d, 10d as ACOT; plus none	(B) Sham acupuncture treatment	21, 3 w follow up	Angina attack rate, NTG consumption and general well-being on an ordinal scale*	Pr4/Se1, 4; Su/Ob
CAO JP 2002 [41]	(A) BA G1 (3 + X)^a^, G2 (3 + X)^a^, alternate G1 and 2 each time, 20 min/d, 15 d as ACOT; plus (B)	(B) ID 5–10 mg Tid, ASP 50–300 mg Tid; BET 25–50 mg Bid and Norvasc 5 mg Qd are chosen according to the disease severity.	15	4 degrees of effects in the symptom; and 3 degrees of effects in the ECG; MID according to DCG	Pr4/Se2; Su
CHANG PF 2005 [42]	(A) BA G1 (4 × 2 + 2), G2 (4 × 2 + 2), alternate G 1 and 2 every other d, 30 min/d for 14 d; plus none	(B) ID 10 mg Tid, or BET 12.5 mg Bid	14	4 degrees of effects in the symptom and ECG; no. Patients of NTG reduction and suspension	Pr4/Se2, 4; Su/Ob
DIAO LH 2006 [43]	(A) EA (1 + X)^a^ 30 min/d, 6 d and 1 d rest as ACOT; plus none	(B) CDP 10 pills (25 mg/pill) Tid	28	4 degrees of effects in the DCG and ECG	Se2, 3; Su
	(A) EA (1 + X)^a^ 30 min/d, 6 d and 1 d rest as ACOT; plus (B)				
HU NK 1997 [44]	(A) BA (1 × 2) 30 min/d, 30 d as ACOT; plus (B)	(B) Huoxinwan (Chinese medicine) 2 pills Bid, compound Danshen 5 tablets Tid, ASP 50 mg Qd	30	3 degrees of effects in the DCG and ECG	Se2, 3; Su
HUANG J 2004 [45]	(A) EA (1 × 2) 30 min/d, 6 d per w for 4 ws; plus none	(B) CDP 10 pills (25 mg/pill) Tid	28	4 degrees of effects in the symptom and ECG; frequency of attacks, NTG consumption	Pr4/Se2, 4; Su/Ob
HUANG J2 2004 [46]	(A) EA (1 × 2) 30 min/d, 6 d and 1 d rest as ACOT; plus none	(B) CDP 10 pills (25 mg/pill) Tid	28	4 degrees of effects in the symptom and ECG	Pr4/Se2; Su
	(A) EA (1 × 2) 30 min/d, 6 d and 1 d rest as ACOT; plus (B)				
LI CP 2005 [47]	(A) BA (10) 25 min/d,12 d and 2 d rest as ACOT; plus (B)	(B) ID, ASP, BET used convention	28	3 degrees of effects in the symptom and ECG	Pr4/Se2; Su
LI HJ 2003 [48]	(A) BA points G1 (3 + X)^a^, points G1 (3 + X)^a^, alternate G 1 and 2 every other d, 20 min/d for 2 w; plus (B)	(B) CDI 20 ml plus 5% GS or 0.9% NS 250 ml i.v.	28	3 degrees of effects in the symptom and ECG	Pr4/Se2; Su
LIU JL 2007 [49]	(A) Needle-embedding (2 × 2), 10–15 min/d, 7 d as ACOT; plus (B)	(B) ISMN (ISMN)20 mg Bid, simvastatin 10 mg Qd, ASP 75–100 mg Qd. SLNTG	14	3 degrees of effects in the symptom and ECG; no. patients of NTG suspension and reduction	Pr4/Se2, 4; Su/Ob
LIU JR 2010 [50]	(A) BA (6) 15–20 min/d, 7 d as ACOT; plus none	(B) CDP 10 pills (25 mg/pill) Tid, SLNTG	28, 3 m fellow up	3 degrees of effects in the symptom and ECG; NTG consumption	Pr4/Se2, 4; Su/Ob
LIU WP 2004 [51]	(A) BA (6) 20 min/d, 13 d and 2 d rest as ACOT; plus (B)	(B) ID 20 mg Bid, Diltiazem hydrochloride 30 mg Tid, ASP 50 mg Qd.	28	3 degrees of effects in the symptom and ECG	Pr4/Se2; Su
LIU WP 2003 [52]	(A) BA (5) 20 min/d, 13 d and 1 d rest as ACOT; plus (B)	(B) Controlled release ID 20 mg Bid.	56	Quality of life	Se1; Su
LIU YF 2012 [53]	(A) BA (5 + x)^a^, 30 min/d; plus none	(B) ID 10 mg Tid, ASP 100 mg Qd, BET 12.5 mg Bid, SLNTG	28	4 degrees of effects in the symptom and ECG	Pr4/Se2; Su
TONG YH 2005 [54]	(A) EA (2 × 2) 20 min/d, 5 d/w for 6 w; plus (B)	(B) ASP 150 mg, ISMN 40 mg Qd, BET 25 mg Bid, Zocor 20 mg Qd, SLNTG	42	EF	Se7; Ob
WANG PJ 2011 [55]	(A) EA (nr) 30 min/d, 6 d/w, alternate bilateral acupoints each time; plus none	(B) CDP 10 pills (25 mg/pill) Tid	28	4 degrees of effects in the DCG	Se3; Su
	(A) EA (nr) 30 min/d, 6 d/w, alternate bilateral acupoints each time; plus (B)				
WANG X 2000 [56]	(A) BA G1 (5), G2 (5), alternate G 1 and 2 in the morning and afternoon from 1 to 5 d, alternate Groups every other day from 6th day to the end, 30 min/d for 14 d; plus (B)	(B) ID or CDI with ASP, NIF, BET	14	3 degrees of effects in the symptom and ECG; duration of angina relief, and angina disappearance	Pr4/Se2; Su/Ob
WU CY 2009 [57]	(A) BA (3 + 1) 30 min/d, 10 d and 2 d rest as ACOT; plus none	(B) CDP 10 pills (25 mg/pill) Tid	34	4 degrees of effects in the symptom; MID according to DCG; no. Patients of NTG reduction and suspension	Pr4/Se3, 4; Su/Ob
WU HH 2005 [58]	(A) BA (10) 30 min/d for 2 w; plus (B)	(B) Nitrates, p.o.; Gegensu injection 500 mg plus 5% GS or 0.85% NS Qd.	14	3 degrees of effects in the symptom; 4 degrees of effects in the ECG	Pr4/Se2; Su
XIE ZQ 2003 [59]	(A) EA (2 × 2) 20–30 min/d, 12 d and 3 d rest as ACOT; plus (B)	(B) ISMN, 10–60 mg, every 4–6 hours; NTG i.v. starts at 5–10 μg/min and increases 5–10 μg per 5 min; NIF 10–20 mg combined with propranlolum 40–80 mg Tid; ASP 0.3-0.6 g Qd; liquaemin injection 5000U, if necessary, 4–6 hours repeated once.	34, a follow up after leaving hospital	Cardiovascular events	Pr3; Ob
XU GD 2006 [60], TONG YH1 2005 [61]	(A) EA (2 × 2) 20 min/d, 5 d and 2 d rest as ACOT; plus (B)	(B) ASP 150 mg Qd, ISMN 40 mg Qd, BET 25 mg, Bid, simvastatin 20 mg Qd, SLNTG	42	Quality of life; 3 degrees of effects in the symptom and ECG; onset time of angina relief, angina disappearance	Pr4/Se1, 2; Su/Ob
YU W 2006 [62]	(A) EA (1 × 2), 30 min/d for 10 d; plus (B)	(B) Atenolol 12.5 mg Bid, ASP 100 mg Qd. SLNTG	10	4 degrees of effects in the symptom and ECG; time of angina attack	Pr4/Se2; Su/Ob
ZHANG L 2011 [63]	(A) BA (6 × 2 + 1) 30 min/d for 4 ws; plus (B)	(B) ASP 100 mg, chiralisomer 75 mg, ISMN 20 mg, Perindopril 2 mg, simvastatin 20 mg Qd	28	3 degrees of effects in the symptom and ECG; EF	Pr4/Se2, 7; Su/Ob
ZHANG LJ 2005 [64]	(A) BA (7) 20 min/d, 10 d and 3 d rest as ACOT; plus none	(B) CDP 10 pills (25 mg/pill) Tid	60	4 degrees of effects in the symptom; 3 degrees of effects in the ECG	Pr4/Se2; Su
	(A) BA (7) 20 min/d, 10 d and 3 d rest as ACOT; plus (B)				

D indicates days; min, minutes; w, week; m, month; BA, Body acupuncture; G, group; EA, electro-acupuncture; ACOT, a course of treatment; ID, Isosorbide dinitrate; CDP, Compound Danshen Pills; ASP, Aspirin; BET, Betaloc; CDI, compound Danshen injection; ISMN, Isosorbide mononitrate; NIF, nifedipine; SLNTG, Sublingual NTG when angina attacks; Pr 4, the 4th primary outcome in the method section, other number as well; Se 2, the 2nd secondary outcome in the method section, other number as well; Su, subjective outcome; Ob, objective outcome; *: much improved, somewhat improved, slightly improved, unchanged, slightly worse, somewhat worse, much worse; MID, myocardial ischemia duration; EF, ejection fraction; ECG: Electrocardiogram; DCG: Dynamic electrocardiogram; Tid: three times daily; Bid: twice daily: Qd: once daily; p.o.: take medicine by oral; i.v.: intravenous injection; NTG: Nitroglycerin; ^a^: The selection of the acupoints according to syndrome differentiation.

Twenty-five trials involving 2058 patients were included. Twenty-three trials were carried out in out/inpatients from Chinese hospitals, and involved a total of 1,983 patients (Treatment Group, TG: 1,098; Control Group, CG: 885) with angina pectoris. The sample size varied from 13 to 120 participants per group, with an average of 36 patients in each group. In total, 696 patients were diagnosed with stable angina (SAP), 289 with unstable angina (UAP), and 998 with coronary heart disease angina pectoris (CHD-AP). A wide variation was noted in the age of the subjects (33–87 years) and in disease duration (2 days-24 years). Twenty-one trials specified seven sets of diagnostic criteria, including five national criteria in China, one from the United States, and one from WHO/ISFC. The other 4 trials [25,36,55,59] used the modified diagnosis criteria (details in Table 1).

**Figure 2:**
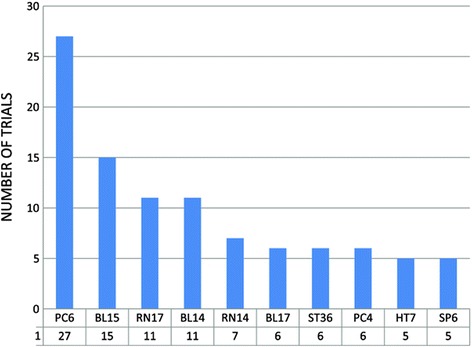
**Top 10 points used for meridian acupuncture treatment.**

Two cases of comparisons have been categorized. One was that acupuncture was compared with sham acupuncture or medicines, and the other was that the combination of acupuncture and non-acupuncture elements (conventional medicines or Chinese patent medicines) was compared with non-acupuncture elements alone. The two trials [35,36] described the sham acupuncture controls and the study characteristics were presented in Additional file 2. The acupoints used in the trials were not fixed and the number of acupoints selected ranged from 1 to 10 (2 trials did not report). The top 10 commonly used body acupoints were presented in Figure 2.

The total treatment duration ranged from 10 days to 60 days. Four trials [35,36,50,59] reported follow up data from 3 weeks to 3 months, but only one trial [36] reported available data for analysis.

None of the trials reported the overall mortality or cardiovascular mortality as outcomes. Twenty trials reported the primary outcomes, where 19 trials reported the functional outcomes and one [59] showed the cardiovascular events. Ejection fraction (EF), angina attack rate, frequency of attacks, duration of angina relief and angina disappearance, nitroglycerin (NTG) consumption, number of patients with NTG reduction and suspension, and quality of life were also outcome measures. The health-related quality of life, ECG, DCG, consumption of existing anti-angina drugs, and EF were reported as the secondary outcomes in four, twenty, four, six, and two trials, respectively. Four trials [35,36,45,62] reported adverse events. Twenty-three subjective measures and 13 objective measures were used as summary outcome measures. All details of the outcome time points were shown in Table 2.

Seventeen trials reported outcome measures according to the Standard on the Assessment of Curative Effect of Angina and ECG of Coronary Heart Disease, constituted by a Symposium on Integrated Western and Chinese Medicine for the Angina of Coronary Heart Disease in 1979, in China; and to the other Standard of guiding clinical research in developing new Traditional Chinese medicine in 1992,2002. They shared the similar criteria (first criterion after the colons, and the other one in the parentheses) as follows.

- “Markedly effective”: angina attack frequency and duration reduce more than 80% at the same level of exercise (angina disappears or basically disappears);
- “Effective”: angina attack frequency and duration reduce between 50% to 80% (angina attacks frequency and duration obviously reduce more than 50%);
- “Ineffective”: angina attack frequency and duration reduce less than 50% (symptom is basically the same as before treatment);
- “Exacerbation”: angina attack frequency and duration increase (angina attack frequency and duration increased or became worse).

Eight trials [41,42,45,46,53,57,62,64] used four-class clinical comprehensive outcome measures including markedly effective, effective, ineffective, and exacerbation; and ten trials [47-51,56,58,60,61,63] used three-class outcome measures: “markedly effective”, “effective” and “ineffective”. Angina pectoris is a disease with the characteristics of exacerbation no matter what interventions are used, be these interventions that delay disease development and/or improve symptoms and quality of life. Because of the variation in outcome measures used and for the purposes of the meta-analysis, “ineffectiveness” of treatment was defined as angina symptoms where there was no change from baseline, where the improvement in symptoms was less than 50% and where symptoms worsened.

Seven trials [42,43,45,46,53,58,62] used a four-class comprehensive effect and 11 trials [41,44,47-51,56,60,61,63] used a three-class outcome measure for changes in ECG. “Number of patients showing no ECG improvement” meant the number of patients whose ECGs were unchanged or worsened compared with ECG tests before treatment. DCG presented either a four-class effective measure in two trials [43,55] or a three-class in one [44], and two trials [41,57] reported the DCG results using myocardial ischemia duration. The “number of patients showing no DCG improvement” shared the same meaning with the ECG ineffectiveness index.

### Methodological quality

The methodological quality [36,41-64] was “high risk of bias” for 24 trials and [35] “unclear risk of bias” for one trial (Additional file 2, Figures 3 and 4). No trial reported sample size calculation. Twelve trials [41-43,46,47,51-53,55,57,59,63] described their randomization procedure, using a random number table, card, or coin tossing. Eight [42,43,51-53,55,57,63] trials were confirmed by calling the authors who had described allocation concealment using opaque envelopes. Five trials [35,36,51,52,55] mentioned a blinding procedure, and two [35,36] clearly reported the blinding to patients and outcome assessors. However, blinding of patients and acupuncturists in the trials was impossible when comparing acupuncture as adjuvant to the medicine versus that medicine alone or comparing acupuncture versus medicine. Therefore, these trials should be rated as high risk of bias for blinding. Three trials [51,52,55] reported blinding to outcome assessors.

**Figure 3:**
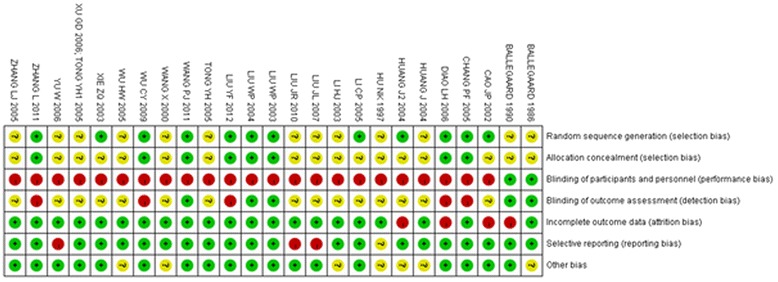
**Risk of bias summary: judgments about each risk of bias item for each study.**

**Figure 4:**
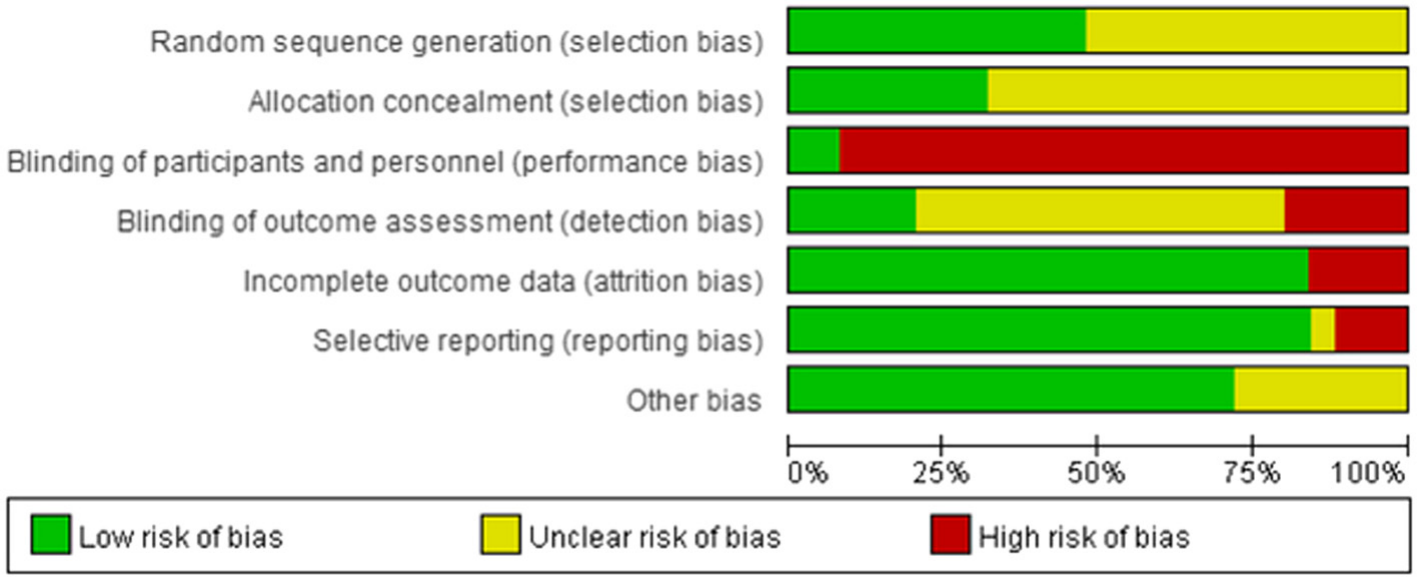
**Risk of bias percentages for each item across all included studies.**

Four trials [35,36,50,59] mentioned follow up, but only one [36] reported available data. Two trials [35,36] reported withdrawal/dropout information, and eight comparisons from four trials [36,41,43,46] were inferred as inconsistent based on the numbers of patients randomized and the numbers analyzed (Table 3). None of the study protocols was accessed, even though seven contacted authors had confirmed that their trials had reported all the outcomes. No trial reported how they dealt with missing data and whether they conducted an intention-to-treat analysis. However, three trials [49,50,62] showed selective reporting because the outcome measures mentioned in the “Methods” section were not reported in the “Results” section. The reporting quality according to the STRICTA (Table 4) varied among the 27 different trials.

**Table 3:** **Trials in which the numbers randomized and the numbers analyzed were inconsistent**

**Study ID**	**Comparison**	**Outcomes**	**TG analysis no./randomized no.**	**CG analysis no./ randomized no.**
BALLEGAARD 1990 [36]	Genuine acupuncture versus sham acupuncture	NTG consumption/ angina attacks	14/24	16/25
CAO JP 2002 [42]	Body acupuncture plus western medicine versus western medicine	DCG	26/30	21/21
DIAO LH 2006 [43]	Electro-acupuncture versus Compound Danshen Pills	ECG	27/30	26/30
DIAO LH (2) 2006 [43]	Electro-acupuncture plus Compound Danshen Pills versus Compound Danshen Pills	ECG	28/30	26/30
HUANG J2 2004 [46]	Electro-acupuncture versus Compound Danshen Pills	ECG	18/20	16/20
HUANG J2 (2) 2004 [46]	Electro-acupuncture plus Compound Danshen Pills versus Compound Danshen Pills	ECG	17/20	16/20

TG: treatment group; CG: controlled group; no.: number; ECG: Electrocardiograph; DCG: Dynamic electrocardiogram; NTG: nitroglycerin.

**Table 4:** Reporting quality of 26 trials according to STRICTA*

**Intervention**	**Description of item**	**No. trials eligible to the principle**	**Percentage**
1 Acupuncture rationale			
	1) Style of acupuncture	25	96.15%
	2) Rationale for treatment (e.g. syndrome patterns, segmental levels, trigger points) and individualization if used	26	92.86%
	3) Literature sources to justify rationale	15	53.57%
2 Needling details		0	
	1) Point used (uni/bilateral)	26	92.86%
	2) Numbers of needles inserted	23	82.14%
	3) Depths of insertion (e.g. cun or tissue level)	16	57.14%
	4) Responses elicited (e.g.de qi or twitch response)	21	75.00%
	5) Needle retention time	20	71.43%
	6) Needle type (gauge, length, and manufacturer or material)	17	60.71%
3 Treatment regimen		0	
	1) Number of treatment sessions	26	92.86%
	2) Frequency of treatment	25	89.29%
4 Co-interventions		0	
	1) Other interventions (e.g. moxibustion, cupping, herbs, exercises, lifestyle advice)	21	75.00%
5 Practitioner background		0	
	1) Duration of relevant training	0	0.00%
	2) Length of clinical experience	18	64.29%
	3) Expertise in specific condition	18	64.29%
6 Control interventions		0	
	1) Intended effect of control intervention and its appropriateness to research question and if appropriate, blinding of participants (e.g. active comparison, minimally active penetrating or nonpenetrating sham, inert)	26	92.86%
	2) Explanations given to patients of treatment and control interventions	2	7.14%
	3) Details of control intervention (precise description, as for item 2 above, and other items if different)	25	89.29%
	4) Sources that justify choice of control	0	0.00%

*, Standards for reporting interventions in controlled trials of acupuncture.

### Effect estimates

All the effect estimates of acupuncture treatment in 25 included trials were listed in the Additional file 3.

#### Primary outcomes

##### Cardiovascular events

One trial [59] with 67 patients reported a significant difference in cardiovascular events when comparing the group receiving a combination of acupuncture and western medicines and the group receiving medicines alone (RR 0.33, 95% CI 0.16-0.65).

### Functional outcomes

#### The number of patients showing ineffectiveness of angina relief

##### Acupuncture versus medicines

Seven trials [42,45,46,50,53,57,64] that included 496 patients compared acupuncture versus medicines. Our meta-analysis revealed no statistical difference between participants who received acupuncture and those who received medicines (RR 0.76, 95% CI 0.53-1.09, details presented in Additional file 4). However, the meta-analysis of two trials [42,53] with 118 patients showed positive effects with acupuncture that were not seen with western medicines such as anti-ischemic and anti-platelet/anti-coagulation therapies (RR 0.39, 95% CI 0.16-0.96). In addition, the meta-analysis of five trials [45,46,50,57,64] with 378 patients showed no statistical difference between acupuncture and Chinese medicines with respect to the number of patients showing ineffectiveness of angina relief (RR 0.86, 95% CI 0.58-1.29).

##### Acupuncture plus other interventions versus other interventions alone

**Figure 5:**
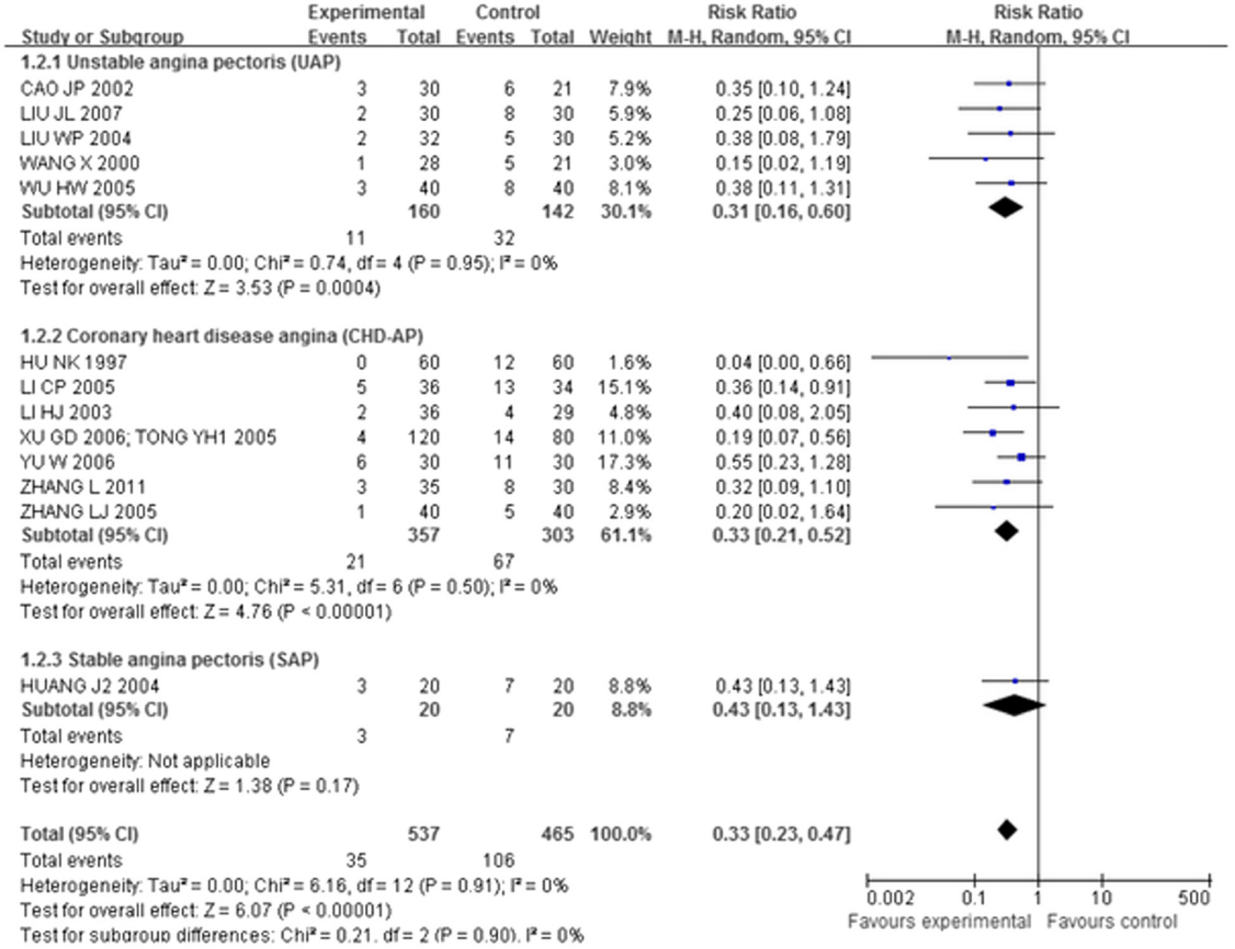
**Overall and subgroup meta-analysis of trials comparing the combination of acupuncture plus other interventions versus other interventions alone in terms of the number of patients showing ineffectiveness of angina relief.** Group 1: unstable angina pectoris; Group 2: coronary heart disease angina; Group3: stable angina pectoris.

**Figure 6:**
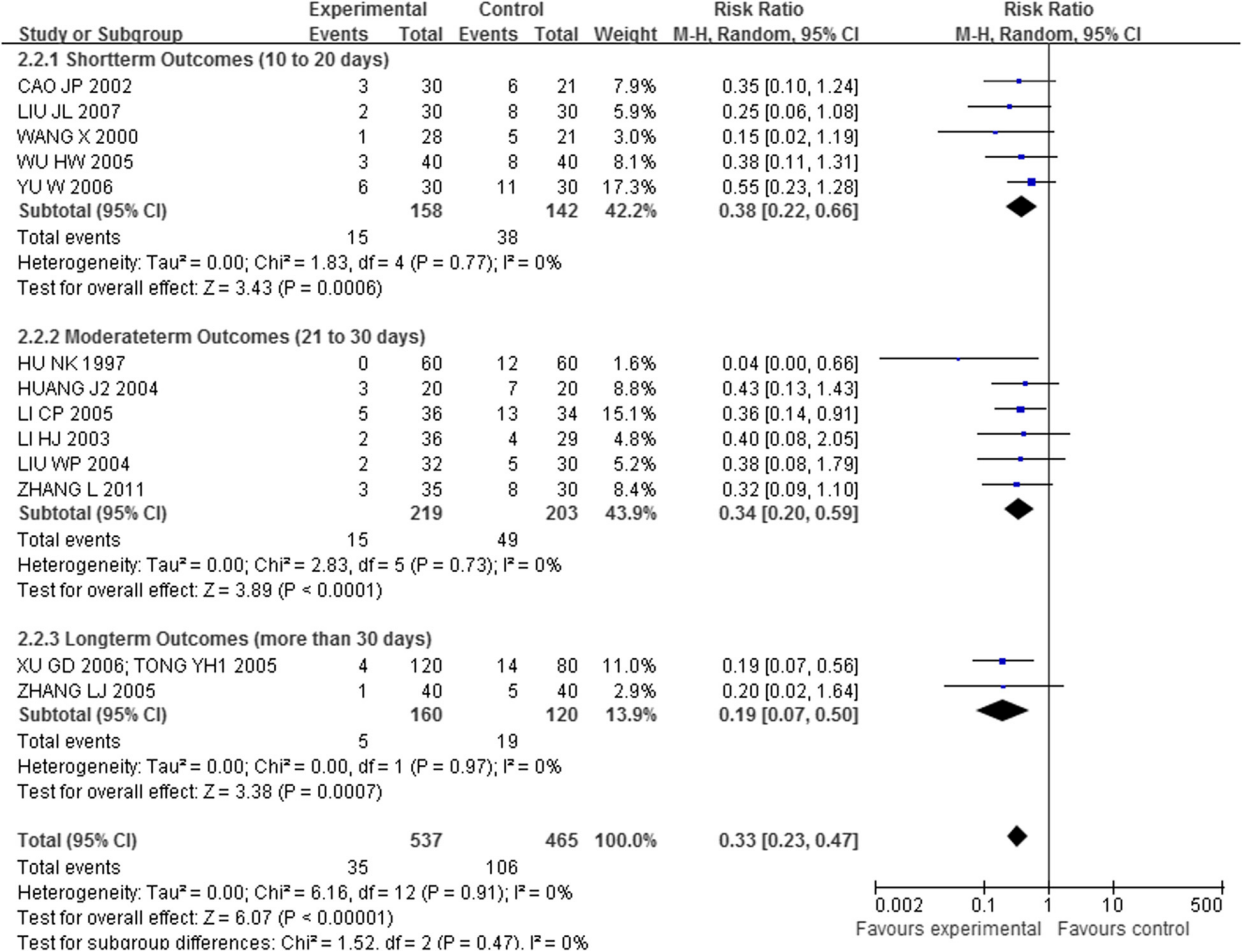
**Overall and subgroup meta-analysis of trials comparing the combination of acupuncture plus other interventions versus other interventions alone in terms of the number of patients showing ineffectiveness of angina relief.** Group 1: short term outcomes; Group 2: moderate term outcomes; Group3: long term outcomes.

**Figure 7:**
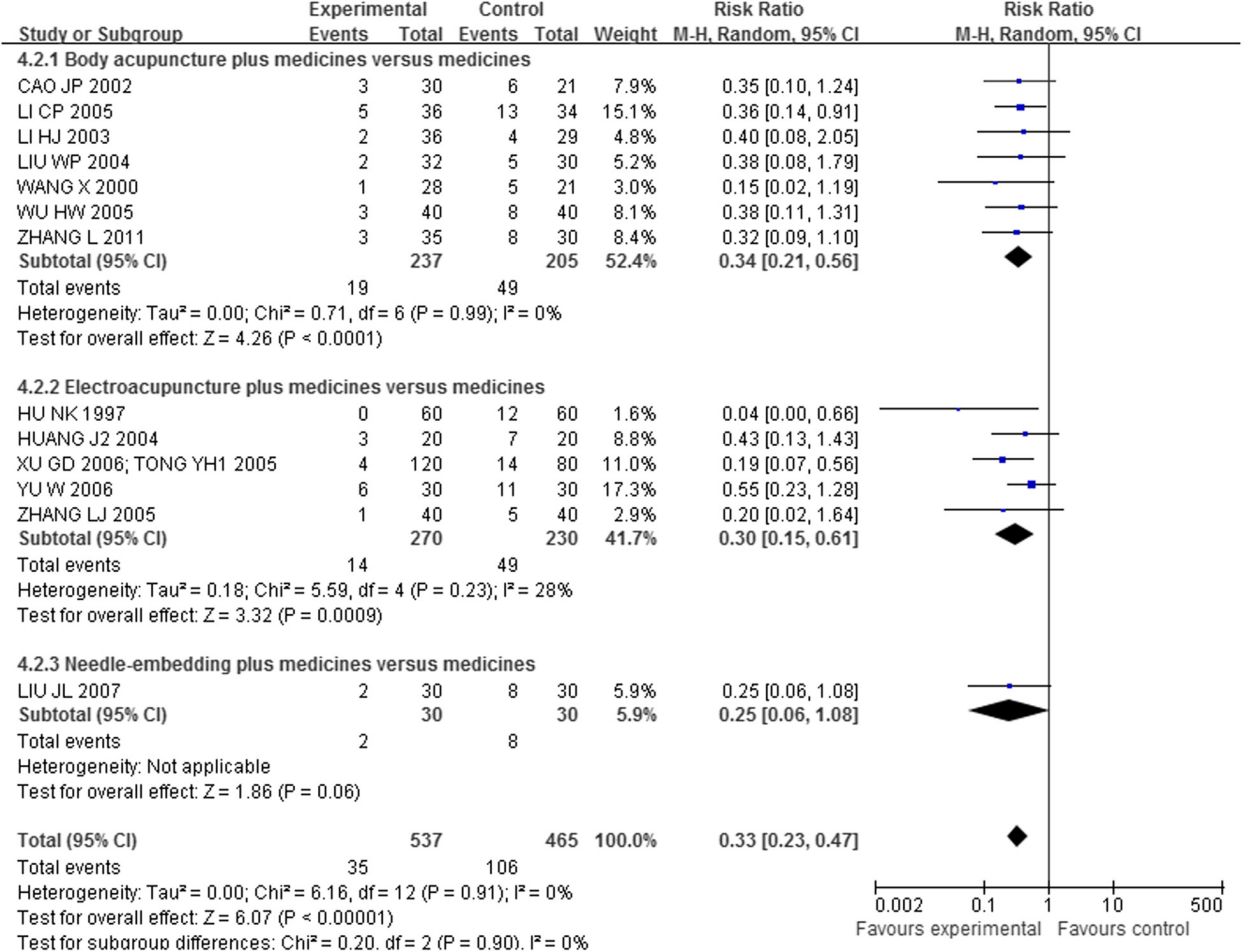
**Overall and subgroup meta-analysis of trials comparing the combination of acupuncture plus other interventions versus other interventions alone in terms of the number of patients showing ineffectiveness of angina relief.** Group 1: Body acupuncture plus medicines versus medicines; Group 2: Electro-acupuncture plus medicines versus medicines; Group3: Needle-embedding plus medicines versus medicines.

Thirteen trials [41,44,46-49,51,56,58,60-64], which included 1,002 patients, compared the effectiveness of acupuncture plus other interventions with the other interventions alone. The meta-analysis results shown in Figures 5, 6, and 7 indicated that the combination of acupuncture and other interventions was more effective than the interventions alone (RR 0.33, 95% CI 0.23-0.47, p < 0.00001, I^2^ = 0%).

The meta-analysis of 7 trials [41,47,49,51,60-63] with 568 patients showed that the combination of acupuncture with western medicines was significantly better than western medicines alone (RR 0.35, 95% CI 0.23-0.53).

The meta-analysis of 3 trials [46,48,64] with 185 patients showed that the combination group of acupuncture and Chinese medicines was significantly better than Chinese medicines alone (RR 0.37, 95% CI 0.15-0.89).

The meta-analysis of 3 trials [44,56,58] with 249 patients showed that the combination of acupuncture with Chinese medicines and western medicines was significantly more effective than the medicines alone (RR 0.20, 95% CI 0.06-0.69).

### Frequency of attacks

#### Acupuncture versus medicines

One trial showed no statistical difference between acupuncture and Chinese medicines with respect to the frequency of attacks [45].

#### Acupuncture plus other interventions versus other interventions alone

One trial [62] showed that the combination of acupuncture and western medicines was more effective than the medicines alone at the frequency of attacks.

### Duration of angina relief and disappearance

One trial [60,61] showed that the combination of acupuncture and western medicines was more effective than western medicines alone at improving the duration of angina relief and promoting disappearance of angina. Another trial [56] also confirmed this result when comparing the combination of acupuncture and western and Chinese medicines with medicines alone.

### Secondary outcomes

#### Quality of life

Two trials [52,60,61] with 263 patients used a total of eight quality of life scales. Meta-analysis of both trials on the symptom, sense of health/happiness and work behaviour showed significant benefit of the combined treatment. Similar meta-analysis results were found for the satisfaction of life, with considerable heterogeneity (I^2^ = 90%), but each trial showed better effect for the combined treatment group. One trial [52] showed no statistical difference in the emotional level between the two groups but meta-analysis of the two trials showed a significant difference in emotion. However, we found no statistical difference with the Basic Activity of Daily Living (BADL), perception of ability, and social status.

### The number of patients showing no ECG improvement

#### Acupuncture versus medicines

Six trials [42,43,45,46,53,64] that included 378 patients compared acupuncture with medicines. No statistical difference was found following meta-analysis of the two groups in terms of the number of patients with no improvement in ECG (RR 0.87, 95% CI 0.65-1.16, details presented in Additional file 5). We also found no statistical difference in two trials [42,53] (118 patients, RR 0.68, 95% CI 0.25-1.84) comparing acupuncture with western medicines or in four trials [43,45,46,64] comparing acupuncture with Chinese medicines (260 patients, RR 0.94, 95% CI 0.70-1.27).

#### Acupuncture plus other interventions versus other interventions alone

**Figure 8:**
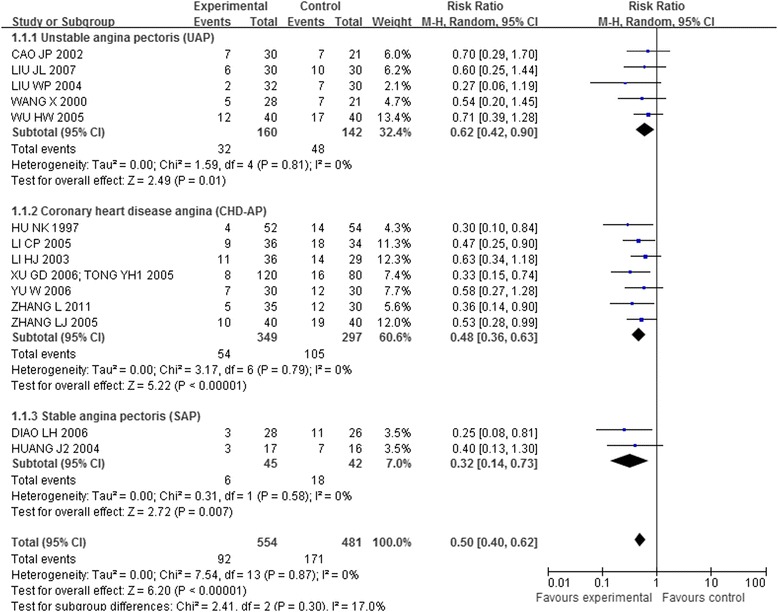
**Overall and subgroup meta-analysis of trials comparing the combination of acupuncture plus other interventions versus other interventions alone in terms of the number of patients showing no ECG improvement.** Group 1: unstable angina pectoris; Group 2: coronary heart disease angina; Group3: stable angina pectoris.

**Figure 9:**
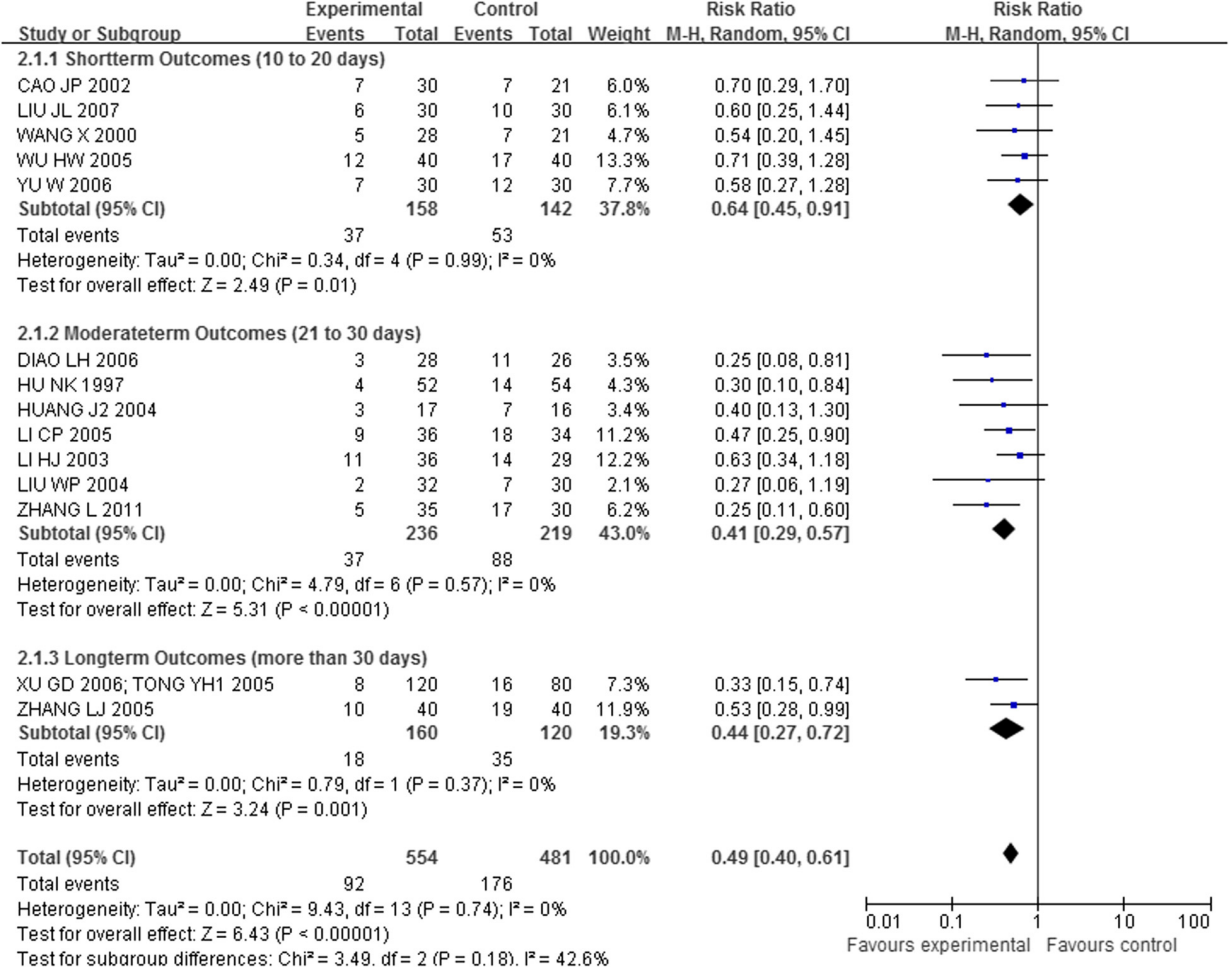
**Overall and subgroup meta-analysis of trials comparing the combination of acupuncture plus other interventions versus other interventions alone in terms of the number of patients showing no ECG improvement.** Group 1: short term outcomes; Group 2: moderate term outcomes; Group3: long term outcomes.

**Figure 10:**
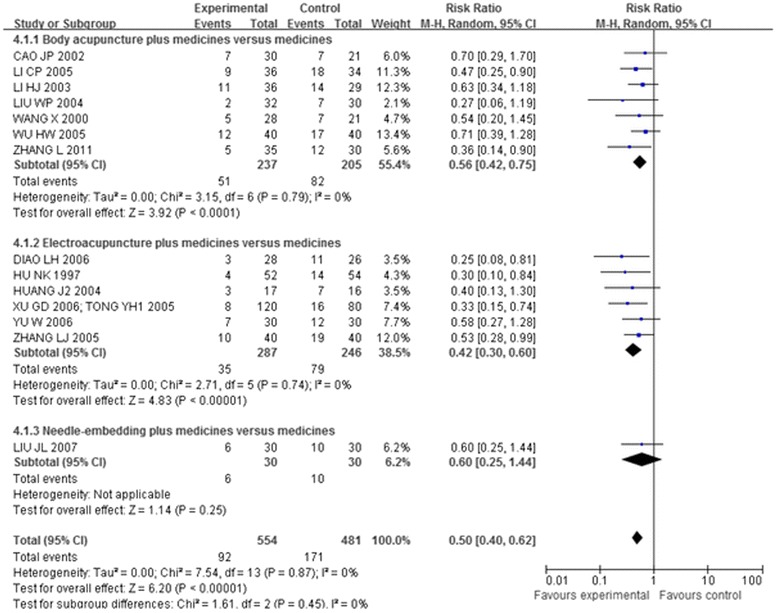
**Overall and subgroup meta-analysis of trials comparing the combination of acupuncture plus other interventions versus other interventions alone in terms of the number of patients showing no ECG improvement.** Group 1: Body acupuncture plus medicines versus medicines; Group 2: Electro-acupuncture plus medicines versus medicines; Group3: Needle-embedding plus medicines versus medicines.

Fourteen trials [41,43,44,46-49,51,56,58,60-64] that included 1,035 patients compared the combination of acupuncture and other interventions with the other interventions alone. The meta-analysis results shown in Figures 8, 9, and 10 indicated that the combination was more effective than the other interventions alone (RR 0.50, 95% CI 0.40-0.62, p < 0.00001, I^2^ = 0%).

The meta-analysis of 7 trials [41,47,49,51,60-63] with 568 patients showed that the combination of acupuncture with western medicines was significantly more effective than the western medicines alone (RR 0.47, 95% CI 0.34-0.65).

The meta-analysis of 4 trials [43,46,48,64] with 245 patients showed that the combination of acupuncture and Chinese medicines was statistically more effective than the Chinese medicines alone (RR 0.51, 95% CI 0.34-0.75).

The meta-analysis of 3 trials [44,56,58] with 249 patients showed that the combination of acupuncture plus Chinese medicines and western medicines group was more effective than the medicines alone (RR 0.56, 95% CI 0.35-0.90).

### DCG

#### Acupuncture versus medicines

Our meta-analysis of 2 trials [43,55] with 60 patients detected no statistical difference between acupuncture and Chinese medicines with the respect to the number of patients showing no DCG improvement (RR 0.68, 95% CI 0.43-1.08) in the treatment of angina pectoris with low heterogeneity.

Another trial [57] also showed no statistical difference between acupuncture and Chinese medicine in terms of the duration of DCG myocardial ischemia.

#### Acupuncture plus other interventions versus other interventions alone

The meta-analysis of 2 trials [43,55] with 120 patients showed that the combination of acupuncture and Chinese medicines was more effective at reducing the number of patients showing no DCG improvement (RR 0.12, 95% CI 0.04-0.36).

However, the trial [41] showed no statistical difference between the combination of acupuncture plus western medicines and western medicines alone in terms of improving the duration of DCG myocardial ischemia.

### NTG consumption, suspension and reduction

#### Acupuncture versus medicines

Two trials [42,57] showed no statistical difference between acupuncture and medicines in terms of the number of patients with NTG suspension and reduction. However, the meta-analysis of 2 trials [45,50] with 142 patients showed positive effects of acupuncture versus Chinese medicines on NTG consumption.

#### Acupuncture plus other interventions versus other interventions alone

One trial [49] showed a positive effect of the combination of acupuncture plus western medicines on the number of patients with NTG suspension and reduction.

### EF

Two trials [54,63] showed that a combination of acupuncture and other medications was more effective at reducing EF, even though the heterogeneity was high (I^2^ = 83%).

### Adverse effect

Adverse events were mentioned in four of the 27 trials [35,36,45,62], comprising 215 patients. Only one trial [45] reported no significant difference between groups receiving electro-acupuncture and Compound Danshen Pills (RR 0.11, 95% CI 0.01 to 2.00, p = 0.92). No complications or adverse effects were observed in the other three trials that included acupuncture treatments.

### Genuine acupuncture versus sham acupuncture

Two trials [35,36] with 75 patients compared genuine acupuncture versus sham acupuncture. The meta-analysis of these two trials indicated no significant difference between the genuine and sham acupuncture in the global evaluation scale after treatment. One trial [36] reported no significant difference in the global evaluation scale after a 3 week follow up, and the number of patients with NTG consumption, or the angina attack rate.

One trial [35] reported the median and range for the angina attack rate [51 (4–194) versus 74 (1–167)] and NTG consumption [27 (0–182) versus 28 (0–161)] after treatment in genuine and sham acupuncture groups. This trial [35] also reported the angina attacks [55 (8–168) versus 66 (41–149)] after a 3 weeks follow up and NTG consumption [39 (1–193) versus 30 (0–152)].

### Subgroup analysis

Fifteen trials [41,43,44,46-49,51,56,58,60-64] compared the combination of acupuncture plus other interventions with other interventions alone on the number of patients who showed ineffectiveness of angina relief and no ECG improvement. The eligible data permitted inclusion only of CHD-AP and UAP in the subgroup analysis (Figures 5 and 8):

The meta-analysis of the UAP (RR 0.31, 95% CI 0.16-0.60; RR 0.62, 95% CI 0.42-0.90) and CHD-AP subgroup (RR 0.33, 95% CI 0.21-0.52; RR 0.48, 95% CI 0.36-0.63) indicated positive effects of combination of acupuncture plus other interventions on the number of patients showing ineffectiveness of angina relief and no improvement in ECG.

Another subgroup analysis of short, moderate, and long treatment durations demonstrated positive effects of the combination of acupuncture plus other interventions on the number of patients showing ineffectiveness of angina relief (RR 0.38,95% CI 0.22-0.66; RR 0.34, 95% CI 0.20-0.59; RR 0.19, 95% CI 0.07-0.50, details presented in Figure 6) and no ECG improvement (RR 0.64, 95% CI 0.45-0.91; RR 0.41, 95% CI 0.29-0.57; RR 0.44, 95% CI 0.27-0.72, details presented in Figure 9).

The third subgroup analysis of body acupuncture and electro-acupuncture (needle-embedding excluded due to insufficient data) showed positive effects of the combination of acupuncture plus other interventions on the number of patients showing ineffectiveness of angina relief (RR 0.34, 95% CI 0.21-0.56; RR 0.30, 95% CI 0.15-0.61, details presented in Figure 7) and no ECG improvement (RR 0.56, 95% CI 0.42-0.75; RR 0.42, 95% CI 0.30-0.60, details presented in Figure 10).

In each of the three subgroup analyses, the confidence intervals overlapped. Thus, no difference has been demonstrated between different durations of treatment, types of angina or types of acupuncture.

### Sensitivity analysis

Sensitivity analyses were robust (see Additional file 6) by excluding unpublished trials, trials with a sample size less than 40 and trials with unclear randomization procedure.

### Publication bias

**Figure 11:**
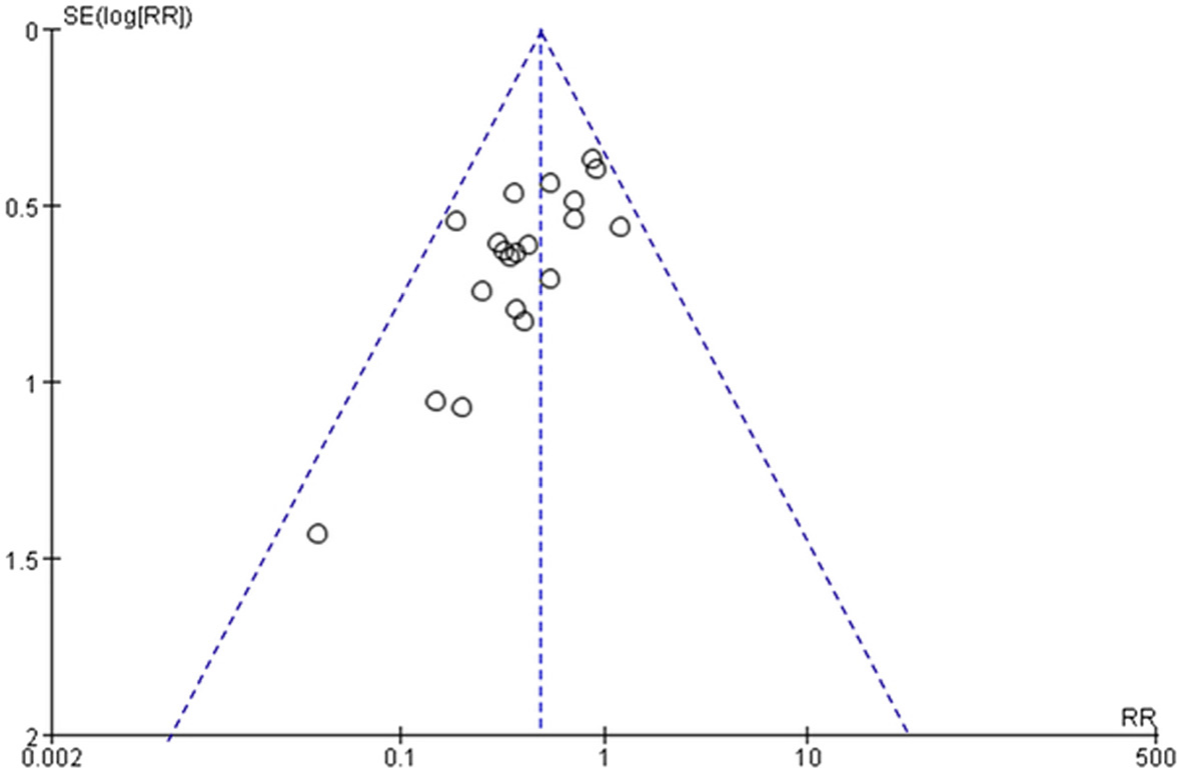
**Funnel plots of 18 trials for the outcome of the number of patients showing ineffectiveness of angina relief.** SE, standard error; RR, relative risk.

A funnel plot analysis was generated for the 18 RCTs comparing acupuncture plus medicines (or none) versus medicines alone with respect to the number of patients showing ineffectiveness of angina relief. The funnel plot of these 18 trials showed asymmetry (see Figure 11).

### Assessment of evidence quality

The “GRADEprofiler” was used to classify the comparisons of the combination of acupuncture with or without medicines and the medicines alone. We found the quality of evidence to be very low (see Table 5).

**Table 5:** Summary of findings*

**Acupuncture for angina pectoris**						
**Patient or population:** patients with angina pectoris						
**Settings:** Mainland China						
**Intervention:** Acupuncture						
**Outcomes**	Illustrative comparative risks* (95% CI)		**Relative effect (95% CI)**	**No of Participants (studies)**	**Quality of the evidence (GRADE)**	**Comments**
	Assumed risk	Corresponding risk				
	**Control**	**Acupuncture**				
**The number of patients showing ineffectiveness of angina relief (acupuncture plus medicines VS medicines)**	**Study population**		**RR 0.35** (0.25 to 0.48)	1002 (13 studies)	⊕⊝⊝⊝ **very low** ^1,2,3,6,7,8,9^	
	**228 per 1000**	**75 per 1000** (52 to 107)				
	**Moderate**					
	**425 per 1000**	**140 per 1000** (98 to 200)				
**The number of patients showing no ECG improvement (acupuncture plus medicines VS medicines)**	**Study population**		**RR 0.50** (0.40 to 0.62)	1035 (14 studies)	⊕⊝⊝⊝ **very low** ^1,2,3,6,7,8^	
	**356 per 1000**	**178 per 1000** (142 to 220)				
	**Moderate**					
	**238 per 1000**	**119 per 1000** (95 to 148)				
**The number of patients showing ineffectiveness of angina relief (acupuncture VS medicines)**	**Study population**		**RR 0.76** (0.53 to 1.09)	516 (7 studies)	⊕⊝⊝⊝ **very low** ^1,5,6,7,8^	
	**216 per 1000**	**164 per 1000** (114 to 235)				
	**Moderate**					
	**182 per 1000**	**138 per 1000** (96 to 198)				
**The number of patients showing no ECG improvement (acupuncture VS medicines)**	**Study population**		**RR 0.87** (0.65 to 1.16)	362 (6 studies)	⊕⊝⊝⊝ **very low** ^1,4,6,7,8^	
	**426 per 1000**	**371 per 1000** (277 to 494)				
	**Moderate**					
	**430 per 1000**	**374 per 1000** (279 to 499)				

*The basis for the assumed risk (e.g. the median control group risk across studies) is provided in footnotes. The corresponding risk (and its 95% confidence interval) is based on the assumed risk in the comparison group and the relative effect of the intervention (and its 95% CI). CI: Confidence interval; RR: Risk ratio;

GRADE Working Group grades of evidence.

High quality: Further research is very unlikely to change our confidence in the estimate of effect.

Moderate quality: Further research is likely to have an important impact on our confidence in the estimate of effect and may change the estimate.

Low quality: Further research is very likely to have an important impact on our confidence in the estimate of effect and is likely to change the estimate.

Very low quality: We are very uncertain about the estimate.

^1^The methodological quality of 24 trials was “high risk of bias” and 1 trials [35] “unclear risk of bias”; due to combining acupuncture treatment plus medicines versus medicines, it is difficult to blind acupuncturists and participants. Nine authors offered the details about the trials, but others have not been contacted;

^2^Inconsistency: Consider the forest plot (Figures 5 and 6), all studies on left side of the line of no effect, where the confidence intervals with minimal overlap, the p value for heterogeneity is greater than 0.05, and I^2^ is 0. All studies favor treatment. The quality of the evidence would not be downgraded for inconsistency based on the fact that the point estimates are compatible with benefit;

^3^large effect: In spite of large effect, the high risk bias threaten the validity;

^4^Inconsistency: Consider the forest plot (Additional file 6), with 6 studies, 4 on left and 2 on right side of the line of no effect, where the confidence intervals overlap, in which the p value for heterogeneity is larger than 0.05, I^2^ is 17%. Heterogeneity could not be explained by study design, differences in population/interventions/outcomes. All studies, except 1 favors treatment. The quality of the evidence would be downgraded for inconsistency;

^5^Inconsistency: Consider the forest plot (Additional file 4), with 7 studies, 5 on left and 1 on right side of the line of no effect, where the confidence intervals overlap, in which the p value for heterogeneity is larger than 0.05, I^2^ is 0%. All studies, except 1 favors treatment. The quality of the evidence would be downgraded for inconsistency. The quality of the evidence would not be downgraded for inconsistency based on the fact that the point estimates are compatible with no effect;

^6^Indirectness: The quality of the evidence may be downgraded when substitute measurements or surrogate endpoints are measured instead of patient-important outcomes, such as mortality, cardiovascular events;

^7^Imprecision: Total number of events is less than 300 (based on: Mueller et al. Ann Intern Med. 2007;146:878–881);95% confidence interval around the pooled effect includes both 1) no effect and 2) appreciable benefit or appreciable harm;

^8^Publication bias: see from funnel plot and potential language bias;

^9^Meta-analysis showed a better effect of acupuncture plus other interventions than other interventions on the outcomes of the number of patients showing ineffectiveness of angina relief and no ECG improvement.

* It shows the quality of evidence for the outcomes of the number of patients showing ineffectiveness of angina relief and no ECG improvement.

The “GRADEprofiler” was used to classify the comparisons of the combination of acupuncture with or without medicines and the medicines alone. We found the quality of evidence to be very low (see Table 5).

## Discussion

The results of the meta-analyses in this paper indicate that the majority of the studies point to a greater effectiveness of the combination of acupuncture with other interventions than the other interventions alone in terms of decreasing the number of patients showing ineffectiveness of angina relief and no ECG improvement. However, we found no statistical difference between participants who underwent acupuncture alone and those who received western medicines or Chinese medicines alone. Therefore, acupuncture might be more effective as an adjuvant treatment with other medicines. The safety of acupuncture for angina needs to be confirmed in future studies because few of the studies reported adverse events, even though another systematic review that examined acupuncture related adverse events for diverse diseases concluded that the acupuncture treatment in the hands of well-trained practitioners was inherently safe, regardless of traumatic, infectious, or other adverse events [65].

A Cochrane systematic review of acupuncture for angina is underway and uses methods that differ slightly from those used in this review. Trials of stimulation of acupuncture points without needling will be included, and trials must involve at least two weeks of treatment and a one month observation period [9].

Another systematic review [66] has published supporting results indicating that the combination of acupuncture with conventional drugs relieved angina symptoms and improved ECG, despite the poor quality of the evidence. However, the evidence presented in a meta-analysis of two low-quality studies is not sufficiently convincing to conclude that acupuncture alone also relieved angina symptoms, improved ECG, and delayed the time of angina onset.

The beneficial effect of acupuncture might also be overestimated because of the small sample size, much heterogeneity, flawed methodology of the included trials, and no consideration of follow-up duration. Several limitations should be considered before accepting the findings of the present paper.

First, the quality of the included trials was compromised by a generally high risk of bias according to the “list of risk” indicated in the Cochrane Handbook [37]. Inadequate reporting of random allocation, allocation concealment, blinding, intention-to-treat analysis, and dropout accounts in the majority of trials may have led to performance bias and detection bias since the patients and researchers were aware of therapeutic interventions for the subjective outcome measures. Most of the trials provided limited descriptions of trial design and the sample size calculation, which precluded estimating the statistical powers; and randomizations were only mentioned without further details, which precluded a proper evaluation of how the trials were conducted. The lack of ITT analysis in the included literature and the meta-analysis indicates the possible presence of selection bias. The funnel plots showing asymmetry also suggested the existence of publication bias because of the low quality and small sample numbers in the trials. In addition, 25 of the included trials were published in Chinese (Table 1), and the other two were published in English, despite searches of Japanese and Korean databases. Most trials were carried out in mainland China, which may be due to the popularity and the lower expense of acupuncture treatment for angina [26], as well as the shortage of medical resources [25]. The sources of recruitment, mainly in the form of hospital outpatient and/or inpatient, also may not be representative of the overall population. Vickers [67] reported that some Asian countries, including China, tend to publish unusually high proportion of positive results. However, the sensitivity analyses illuminated the robustness of the results, when unpublished trials, less sample size, and unclear randomization procedure were taken into consideration.

The clinical heterogeneity could be due to the extent of the variations among the trials in aspects such as participants, diagnosis criteria, intervention, control group, treatment duration, and loss of ITT analysis. The trials used different diagnostic criteria for angina. The patients were heterogeneous in terms of age, disease duration, complications, and types of angina. The subgroup analysis showed similar effects in CHD-AP and UAP patients when treated by combination of acupuncture and medications. However, subgroup analysis of angina severity should be conducted using the classifications recommended by the Canadian Cardiovascular Society Classification (CCSC) or the New York Heart Association (NYHA), but this was not done in any of the published trials. Therefore, we recommended that future trials analyze the differences among the CCSC or NYHA classifications with respect to angina treatments.

The heterogeneity of the acupuncture results may reflect the different kinds of acupuncture treatments used (one trial used needle-embedding, nine used electro-acupuncture, fifteen used body acupuncture). Eighteen trials included the combination of acupuncture with medicines. Even though much heterogeneity existed, in the subgroup analyses, the combinations of body needling or electro-acupuncture and medications treatment were more beneficial than medications alone.

Four trials selected acupoints according to the TCM syndrome differentiation based on Chinese medicine theory, whereas the other 21 trials used fixed acupoints throughout the treatment. The acupoints were selected from one to ten in the acupuncture treatment trials. The controls for these trials were also heterogeneous and included Western medicines, Chinese medicines (mainly Compound Danshen pill), western and Chinese medicines and sham acupuncture. The treatment durations varied from 10 to 60 days. However, the subgroup analysis of treatment durations showed the combination group had better effect no matter how long patients had been treated.

The use of comprehensive effect in 20 trials to evaluate the number of patients showing ineffectiveness of angina relief or no ECG or DCG improvement also limits the generalization of the findings. The classifications of markedly effective, effective, ineffective and/or exacerbation are not internationally recognized, and complicates the interpretation of the effects. In addition, the researchers always selected three or four categories from criteria for outcome evaluation, making it difficult to conduct the meta-analysis. Above all, reaching a consensus for the rationale for the dealing with dichotomising data from three or four categories appeared to be difficult and usually required discussion with clinical experts.

Meanwhile, four meta-analyses showed considerable statistical heterogeneity with respect to angina attacks, EF, satisfaction of life, and global evaluation after treatment. The clinical heterogeneity requires that the interpretation of the positive findings from the meta-analyses be incorporated with the clinical characteristics of the included trials and evidence strength.

Finally, according to the STRICTA (Table 4), more than 80% of the trials reported the style of acupuncture, rationale for treatment, point used, number of needles inserted, number of treatment sessions, frequency of treatment, co-interventions, and control interventions (intended effect and appropriateness and details of control intervention), but only about 50% of the trials reported needling techniques such as the depth of insertion, responses elicited, needle retention time and needle type, and less than 30% of the trials mentioned the details of control intervention (explanations to patients and Sources of control subjects) and practitioner’s background. Therefore, trials should be reported according to the STRICTA statement [38].

## Conclusion

In conclusion, our review indicates that a combination of acupuncture plus medicines may improve angina symptom, ECG, DCG and quality of life; but the safety of acupuncture for angina needs to be confirmed in future studies. However, this systematic review still did not reveal conclusive benefits for acupuncture, with the quality of evidence judged as very low by GRADE evaluation, due to the limitations of the included trials stated above. Further rigorous, high-quality trials with larger sample sizes are needed to confirm the effectiveness of acupuncture in treating angina. Randomization methods need to be clearly described and fully reported. Although blinding of the acupuncturists might be very difficult [68], attempts should be made to the extent possible to blind the outcome assessors and keep data inaccessible to practitioners in order to minimize performance and assessment biases [69].

Analysis of outcomes based on intention-to-treat principle is important. Coronary heart disease consists of stable and unstable angina with different disease durations and severity; therefore, acupuncture is likely to have different effects on different subgroups of patients. Future clinical trials should focus on particular subgroups or include a very large sample size in order to delineate the effect of acupuncture on the different types of patients or different treatment techniques. In addition, well-defined diagnostics should be employed to make a precise clinical diagnosis of angina, thereby increasing the comparability between trials. We also recommended that future trials comply with international standards in the evaluation of treatment effects.

Reporting of trials should follow the Consolidated Standards of Reporting Trials [70] to explicitly explain the process of the treatment, so that other clinicians or researchers can possibly use or replicate this process. The acupuncturist’s technical competence may influence the therapeutic effect; therefore, we suggest that researchers describe their treatment in detail and that future trials be conducted by qualified, well-trained acupuncturists according to the STRICTA guidelines [38]. Angina is a life-long disease, so a longer follow-up period with serial measurement of outcomes is important to determining the effectiveness and long-term benefits of acupuncture.

## Additional files

Additional file 1:
**The search strategies of electronic database.**
Additional file 2:
**The risk of bias contents of the included trails.**
Additional file 3:
**Effect estimates of acupuncture treatment in 25 included trials.**
Additional file 4:
**Meta-analyses of trials comparing acupuncture verse medicines in the no. patients with ineffectiveness of angina symptom.**
Additional file 5:
**Meta-analyses of trials comparing acupuncture verse medicines in the no. patients with ineffectiveness of ECG.**
Additional file 6:
**Summary table of Sensitive analyses.**
