# Supplementary material for: Effectiveness of acupuncture for angina pectoris: a systematic review of randomized controlled trials
Source: BMC Complement Altern Med. 2015 Mar 28;15:90. doi: 10.1186/s12906-015-0586-7 (PMC4426772; doi:10.1186/s12906-015-0586-7)
Supplement: Additional file 1: — The search strategies of electronic database. [file 12906_2015_586_MOESM1_ESM.docx]

**Additional file 1 The search strategies of electronic database**

**MEDLINE**

#1 angina pectoris.mt.; #2 angina*.tw.; #3 angor*.tw.; #4 chest near pain*.tw.; #5 or/1-4; #6 acupuncture therapy.mt.; #7 acupunctur*.tw.; #8 needling or manual acupuncture.tw.; #9 pharmacoacupuncture*.tw.; #10 pharmaco-acupuncture*.tw.; #11 electroacupunctur*.tw.; #12 electro-acupunctur*.tw.; #13 auricular acupuncture*.tw.; #14 ear acupuncture*.tw.; #15 acupoint*.tw.; #16 trigger point*.tw.; #17 meridians or acupuncture points or acupoints.tw.; #18 acupuncture points.tw.; #19 acupoints.tw.; #20 or/6-19; #21 5 and 20.

**EMBASE**

#1 'angina pectoris'/exp OR angina* OR angor* OR ('chest'/de AND near AND pain*) AND [humans]/lim AND ([embase]/lim OR [embase classic]/lim); #2 electro AND near AND acupunctur* OR auricular AND acupuncture* OR 'ear'/de AND acupuncture* OR acupoint* OR trigger AND point* OR 'meridians'/de OR 'acupuncture'/de AND points OR acupoints OR 'acupuncture'/de AND points OR acupoints or pharmaco AND near AND acupuncture* OR electroacupunctur* or 'acupuncture therapy'/exp OR acupunctur* OR needling OR manual AND 'acupuncture'/de OR pharmacoacupuncture* AND [humans]/lim AND ([embase]/lim OR [embase classic]/lim); #3 1and 2

**Cochrane library**

Angina was searched in the Cochrane disc and then viewed the articles about acupuncture for angina.

**CNKI (in English and Chinese)**

(su=angina pectoris + chest near pain* or ft= angina pectoris + chest near pain*) and (su=acupuncture therapy + acupunctur*+ needling + manual acupuncture + pharmacoacupuncture + pharmaco-acupuncture + electroacupunctur* + electro-acupunctur* + auricular acupuncture* + ear acupuncture* + acupoint* + trigger point* + meridians + acupuncture points + acupoints + acupuncture points + acupoints or ft=acupuncture therapy + acupunctur*+ needling + manual acupuncture + pharmacoacupuncture + pharmaco-acupuncture + electroacupunctur* + electro-acupunctur* + auricular acupuncture* + ear acupuncture* + acupoint* + trigger point* + meridians + acupuncture points + acupoints + acupuncture points + acupoints)

(SU=心绞痛+胸痛 or ft=心绞痛+胸痛+心痛+胸痹) and (SU=针刺疗法+针药并用 or ft=针灸+针刺+电针+手捻针+针药+激光针刺+手捻针 or ft=穴位+敏感点+经络)

**WangFang (in English and Chinese)**

(keywords=angina pectoris or chest near pain*) and (keywords=acupuncture therapy or acupunctur* or needling or manual acupuncture or pharmacoacupuncture or pharmaco-acupuncture or electroacupunctur* or electro-acupunctur* or auricular acupuncture* or ear acupuncture* or acupoint* or trigger point* or meridians or acupuncture points or acupoints or acupuncture points or acupoints)

(keywords="心绞痛" or "胸痛" or "心痛" or "胸痹") and (keywords="针刺疗法" or "针药并用" or "针灸" or "针刺" or "电针" or "手捻针" or "针药" or "激光针刺" or "手捻针" or "穴位" or "敏感点" or "经络")

**CBM (in English and Chinese)**

#1 mt= angina pectoris or chest near pain*; #2 kw= angina pectoris or chest near pain*; #3 (#1) or (#2); #4 mt= pharmacoacupuncture or acupuncture therapy; #5 kw=acupuncture therapy or acupunctur* or needling or manual acupuncture or pharmacoacupuncture or pharmaco-acupuncture or electroacupunctur* or electro-acupunctur* or auricular acupuncture* or ear acupuncture* or acupoint* or trigger point* or meridians or acupuncture points or acupoints or acupuncture points or acupoints; #6 (#4) or (#5); #7 (#3) and (#6)

#1主题词="心绞痛" or "胸痛"; #2关键词="心绞痛" or "胸痛" or "心痛" or "胸痹"; #3 (#1) or (#2); #4主题词="针刺疗法" or "针药并用"; #5关键词="针灸" or "针刺" or "电针" or "手捻针" or "针药" or "激光针刺" or "手捻针" or "穴位" or "敏感点" or "经络"; #6 (#4) or (#5); #7 (#3) and (#6);

**VIP (in English and Chinese)**

(ti=angina pectoris + chest near pain* or kw= angina pectoris + chest near pain*) and (ti=acupuncture therapy + acupunctur* + needling + manual acupuncture + pharmacoacupuncture + pharmaco-acupuncture + electroacupunctur* + electro-acupunctur* + auricular acupuncture* + ear acupuncture* + acupoint* + trigger point* + meridians + acupuncture points + acupoints + acupuncture points + acupoints or kw=acupuncture therapy + acupunctur*+ needling + manual acupuncture + pharmacoacupuncture + pharmaco-acupuncture + electroacupunctur* + electro-acupunctur* + auricular acupuncture* + ear acupuncture* + acupoint* + trigger point* + meridians + acupuncture points + acupoints + acupuncture points + acupoints)

(Title=心绞痛+胸痛+心痛+胸痹 or keyword=心绞痛+胸痛+心痛+胸痹) and (Title=针刺疗法+针药并用+激光针刺+针灸+针刺+电针+手捻针+针药+穴位+敏感点+经络or keyword=针刺疗法+针药并用+激光针刺+针灸+针刺+电针+手捻针+针药+穴位+敏感点+经络)

**Korean databases: KISTI-DB, NDSL and KISS (in English and Korean)**

Tree words angina and acupuncture were used for the database searching.

침구 and 협심증

**Japanese databases: ICHUSHI (in English and Japanese)**

Tree words angina and acupuncture were used for the database searching.

狭心症 and鍼治療
