# Supplementary material for: Effectiveness of acupuncture for angina pectoris: a systematic review of randomized controlled trials
Source: BMC Complement Altern Med. 2015 Mar 28;15:90. doi: 10.1186/s12906-015-0586-7 (PMC4426772; doi:10.1186/s12906-015-0586-7)
Supplement: Additional file 2: — The risk of bias contents of the included trails. [file 12906_2015_586_MOESM2_ESM.docx]

**Attachment 2 The risk of bias contents of the included trails**

| **Study ID** | **RANDOM SEQUENCE** | **ALLOCATION CONCEALMENT** | **BLINDING OF PARTICIPANTS** | **BLINDING OF OUTCOME ASSESSMENT** | **INCOMPLETE OUTCOME DATA** | **SELECTIVE REPORTING** | **OTHER BIAS^#^** |
| --- | --- | --- | --- | --- | --- | --- | --- |
| **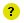**  **BALLEGAARD 1986** | 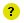  Unclear only based on "randomized into 2 groups"; author was not contacted. | 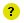  No mention was in the paper; and the author was not contacted. | 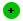  "Sham treatment: needles were inserted superficially through the skin, with no attempt to obtain needle sensation, in points with the same spinal segments as the acupoints, but outside the Chinese meridian system and not at trigger points. The needles were then left untouched. The treatment was carried out in the hospital on an out-patient basis." | 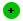  "The evaluation was carried out by the other authors on a blind basis." | 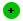  Based on checking the number of patients in Analysis section and randomized number, there might be no missing data. | 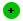  Based on checking the outcome measures in Analysis and Method sections, there might be no selective report. | 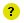  Unclear due to lack of eligible information. |
| **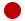**  **BALLEGAARD 1990** | 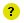  Unclear only based on "randomized into 2 groups"; author was not contacted. | 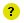  No mention was in the paper; and the author was not contacted. | 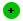  "In the control, needles were inserted superficially through the skin, with no attempt to obtain needle sensation, in points with the same spinal segments as the acupoints, but outside the Chinese meridian system and not at trigger points. The needles were then left untouched. The treatment was carried out in the hospital on an out-patient basis. All patients were told that they were receiving genuine acupuncture, and that the study was a comparison between two different kinds of acupuncture. To increase the patients' confidence that they were receiving the correct acupuncture treatment, the acupuncturist employed an electrically-resistant measurement device, which was adjusted to beep over both genuine and sham points. He then explained to the patient that the beep indicated the exact location of the acupoint and would then confirm the accuracy required for correct needle technique." | 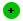  "The evaluation was carried out by the other authors on a blind basis." | 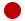  The number of patients in the "Nitroglycerin consumption" and "angina attack rate" of Analysis section disaccorded with randomized number, there was missing data. And there was no ITT analysis published in the paper. | 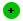  Based on checking the outcome measures in Analysis and Method sections, there might be no selective report. | 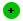  It seemed to be true that article said "Baseline of participants was balanced." And others are probably suitable to low risk. |
| **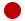**  **CAO JP 2002** | 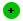  "Tossing a coin was used to randomize the patients into two groups" | 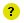  No mention was in the paper; and the author was not contacted. | 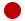  The trials compared acupuncture as adjuvant to medicine versus that medicine alone or compared acupuncture versus medicine. It was impossible to blind participants and acupuncturists. | 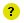  No mention was in the paper; and the author was not contacted. | 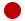  The number of patients in the "DCG myocardial ischemia duration" of Analysis section disaccorded with randomized number, there was missing data. And there was no ITT analysis published in the paper. | 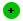  We could not check whether there was a selective report due to combination of Method section and Analysis section in paper. | 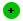  It seemed to be true that article said "Baseline of participants was balanced." And others are probably suitable to low risk. |
| **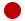**  **CHANG PF 2005*** | 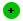  The author told that Random number table was used to randomize the patients into 2 groups. | 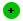  The author told that Opaque envelope was used to do the allocation concealment. | 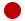  The author told there was no blinding on patients in the trials. | 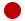  The author told there was no blinding on outcomes assessors in the trials. | 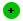  Based on checking the number of patients in Analysis section and randomized number, there might be no missing data. And so the author confirmed. | 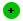  Based on checking the outcome measures in Analysis and Method sections, there might be no selective report. And so the author confirmed. | 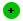  It seemed to be true that article said "Baseline of participants was balanced." And others are probably suitable to low risk. And so the author confirmed. |
| **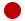**  **DIAO LH 2006*** | 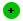  The author told that Random number table was used to randomize the patients into 3 groups. | 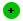  The author told that Opaque envelope was used to do the allocation concealment. | 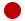  The author told there was no blinding on patients in the trials. | 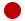  The author told there was no blinding on outcomes assessors in the trials. | 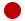  The number of patients in ECG Analysis section of 3 groups disaccorded with randomized number, there was missing data. And there was no ITT analysis published in the paper. | 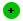  Based on checking the outcome measures in Analysis and Method sections, there might be no selective report. And so the author confirmed. | 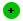  It seemed to be true that article said "Baseline of participants was balanced." And others are probably suitable to low risk. And so the author confirmed. |
| **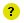**  **HU NK 1997** | 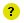  Unclear only based on "randomized into 2 groups"; author was not contacted. | 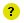  No mention was in the paper; and the author was not contacted. | 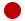  The trials compared acupuncture as adjuvant to medicine versus that medicine alone or compared acupuncture versus medicine. It was impossible to blind participants and acupuncturists. | 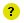  No mention was in the paper; and the author was not contacted. | 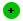  Based on checking the number of patients in Analysis section and randomized number, there might be no missing data. | 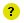  We could not check whether there was a selective report due to combination of Method section and Analysis section in paper. | 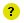  Unclear due to lack of eligible information. |
| **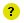**  **HAUNG J 2004** | 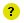  Unclear only based on "randomized into 2 groups"; author was not contacted. | 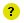  No mention was in the paper; and the author was not contacted. | 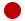  The trials compared acupuncture as adjuvant to medicine versus that medicine alone or compared acupuncture versus medicine. It was impossible to blind participants and acupuncturists. | 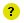  No mention was in the paper; and the author was not contacted. | 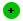  Based on checking the number of patients in Analysis section and randomized number, there might be no missing data. | 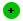  Based on checking the outcome measures in Analysis and Method sections, there might be no selective report. | 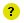  Unclear due to lack of eligible information. |
| **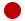**  **HUANG J2 2005** | 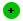  "Random number table was used to randomize the patients into 3 groups." | 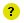  No mention was in the paper; and the author was not contacted. | 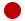  The trials compared acupuncture as adjuvant to medicine versus that medicine alone or compared acupuncture versus medicine. It was impossible to blind participants and acupuncturists. | 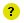  No mention was in the paper; and the author was not contacted. | 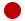  The number of patients in ECG Analysis section of 3 groups disaccorded with randomized number, there was missing data. And there was no ITT analysis published in the paper. | 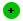  Based on checking the outcome measures in Analysis and Method sections, there might be no selective report. | 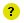  Unclear due to lack of eligible information. |
| **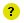**  **LI CP 2005** | 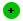  "Random card was used to randomize the patients into 2 groups." | 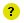  No mention was in the paper; and the author was not contacted. | 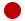  The trials compared acupuncture as adjuvant to medicine versus that medicine alone or compared acupuncture versus medicine. It was impossible to blind participants and acupuncturists. | 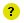  No mention was in the paper; and the author was not contacted. | 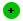  Based on checking the number of patients in Analysis section and randomized number, there might be no missing data. | 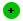  Based on checking the outcome measures in Analysis and Method sections, there might be no selective report. | 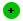  It seemed to be true that article said "Baseline of participants was balanced." And others are probably suitable to low risk. |
| **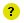**  **LI HJ 2003** | 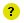  Unclear only based on "randomized into 2 groups"; author was not contacted. | 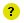  No mention was in the paper; and the author was not contacted. | 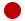  The trials compared acupuncture as adjuvant to medicine versus that medicine alone or compared acupuncture versus medicine. It was impossible to blind participants and acupuncturists. | 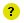  No mention was in the paper; and the author was not contacted. | 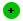  Based on checking the number of patients in Analysis section and randomized number, there might be no missing data. | 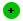  Based on checking the outcome measures in Analysis and Method sections, there might be no selective report. | 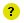  Unclear due to lack of eligible information. |
| **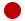**  **LIU JL 2007** | 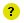  Unclear only based on "randomized into 2 groups"; author was not contacted. | 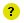  No mention was in the paper; and the author was not contacted. | 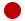  The trials compared acupuncture as adjuvant to medicine versus that medicine alone or compared acupuncture versus medicine. It was impossible to blind participants and acupuncturists. | 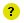  No mention was in the paper; and the author was not contacted. | 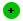  Based on checking the number of patients in Analysis section and randomized number, there might be no missing data. | 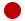  The safety related outcome measures ALT, BUN in Method section were not reported in Analysis section. | 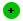  It seemed to be true that article said "Baseline of participants was balanced." And others are probably suitable to low risk. |
| **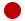**  **LIU JR 2010** | 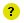  Unclear only based on "randomized into 2 groups"; author was not contacted. | 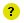  No mention was in the paper; and the author was not contacted. | 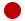  The trials compared acupuncture as adjuvant to medicine versus that medicine alone or compared acupuncture versus medicine. It was impossible to blind participants and acupuncturists. | 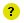  No mention was in the paper; and the author was not contacted. | 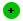  Based on checking the number of patients in Analysis section and randomized number, there might be no missing data. | 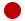  The outcome measure "angina relapse in 3 month" in Method section was not reported in Analysis section. | 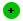  It seemed to be true that article said "Baseline of participants was balanced." And others are probably suitable to low risk. |
| **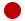**  **LIU WP 2004^*^** | 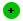  "Random number table was used to randomize the patients into 2 groups." | 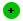  Opaque envelope | 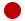  The author told that there was no blinding of patients in the trials. | The author told that the blinding of outcome assessment was done in the trials. | Based on checking the number of patients in Analysis section and randomized number, there might be no missing data. | The author told there was no selecting reporting in trial. | It seemed to be true that article said "Baseline of participants was balanced." And others are probably suitable to low risk. |
| **LIU WP 2003^*^** | "Random number table was used to randomize the patients into 2 groups." | "Opaque envelope was used to do the allocation concealment." | The author told that there was no blinding of patients in the trials. | The author told that the blinding of outcome assessment was done in the trials. | Based on checking the number of patients in Analysis section and randomized number, there might be no missing data. | The author told there was no selecting reporting in trial. | It seemed to be true that article said "Baseline of participants was balanced." And others are probably suitable to low risk. |
| **LIU YF 2012^*^** | The author told that Random number table was used to randomize the patients into 2 groups. | The author told that Opaque envelope was used to do the allocation concealment. | The author told there was no blinding on patients in the trials. | The author told there was no blinding on outcomes assessors in the trials. | Based on checking the number of patients in Analysis section and randomized number, there might be no missing data. | The author told there was no selecting reporting in trial. | It seemed to be true that article said "Baseline of participants was balanced." And others are probably suitable to low risk. |
| **TONG YH 2005** | Unclear only based on "randomized into 2 groups"; author was not contacted. | No mention was in the paper; and the author was not contacted. | The trials compared acupuncture as adjuvant to medicine versus that medicine alone or compared acupuncture versus medicine. It was impossible to blind participants and acupuncturists. | No mention was in the paper; and the author was not contacted. | Based on checking the number of patients in Analysis section and randomized number, there might be no missing data. | Based on checking the outcome measures in Analysis and Method sections, there might be no selective report. | It seemed to be true that article said "Baseline of participants was balanced." And others are probably suitable to low risk. |
| **WANG PJ 2011*** | The author told that Random number table was used to randomize the patients into 3 groups. | The author told that Opaque envelope was used to do the allocation concealment. | The author told there was no blinding on patients in the trials. | The author told that the blinding of outcome assessment was done in the trials. | Based on checking the number of patients in Analysis section and randomized number, there might be no missing data. | The author told there was no selecting reporting in trial. | It seemed to be true that article said "Baseline of participants was balanced." And others are probably suitable to low risk. |
| **WANG X 2000** | Unclear only based on "randomized into 2 groups"; author was not contacted. | No mention was in the paper; and the author was not contacted. | The trials compared acupuncture as adjuvant to medicine versus that medicine alone or compared acupuncture versus medicine. It was impossible to blind participants and acupuncturists. | No mention was in the paper; and the author was not contacted. | Based on checking the number of patients in Analysis section and randomized number, there might be no missing data. | Based on checking the outcome measures in Analysis and Method sections, there might be no selective report. | Unclear due to lack of eligible information. |
| **WU CY 2009*** | The author told that Random number table was used to randomize the patients into 2 groups. | The author told that Opaque envelope was used to do the allocation concealment. | The author told there was no blinding on patients in the trials. | The author told there was no blinding on outcomes assessors in the trials. | Based on checking the number of patients in Analysis section and randomized number, there might be no missing data. | The author told there was no selecting reporting in trial. | It seemed to be true that article said "Baseline of participants was balanced." And others are probably suitable to low risk. |
| **WU HW 2005** | Unclear only based on "randomized into 2 groups"; author was not contacted. | No mention was in the paper; and the author was not contacted. | The trials compared acupuncture as adjuvant to medicine versus that medicine alone or compared acupuncture versus medicine. It was impossible to blind participants and acupuncturists. | No mention was in the paper; and the author was not contacted. | Based on checking the number of patients in Analysis section and randomized number, there might be no missing data. | Based on checking the outcome measures in Analysis and Method sections, there might be no selective report. | Unclear due to lack of eligible information. |
| **XIE ZQ 2003** | "Random number table was used to randomize the patients into 2 groups." | No mention was in the paper; and the author was not contacted. | The trials compared acupuncture as adjuvant to medicine versus that medicine alone or compared acupuncture versus medicine. It was impossible to blind participants and acupuncturists. | No mention was in the paper; and the author was not contacted. | Based on checking the number of patients in Analysis section and randomized number, there might be no missing data. | Based on checking the outcome measures in Analysis and Method sections, there might be no selective report. | It seemed to be true that article said "Baseline of participants was balanced." And others are probably suitable to low risk. |
| **XU GD 2006, TONG YH1 2005** | Unclear only based on "randomized into 2 groups"; author was not contacted. | No mention was in the paper; and the author was not contacted. | The trials compared acupuncture as adjuvant to medicine versus that medicine alone or compared acupuncture versus medicine. It was impossible to blind participants and acupuncturists. | No mention was in the paper; and the author was not contacted. | Based on checking the number of patients in Analysis section and randomized number, there might be no missing data. | Based on checking the outcome measures in Analysis and Method sections, there might be no selective report. | It seemed to be true that article said "Baseline of participants was balanced." And others are probably suitable to low risk. |
| **YU W 2006** | Unclear only based on "randomized into 2 groups"; author was not contacted. | No mention was in the paper; and the author was not contacted. | The trials compared acupuncture as adjuvant to medicine versus that medicine alone or compared acupuncture versus medicine. It was impossible to blind participants and acupuncturists. | No mention was in the paper; and the author was not contacted. | Based on checking the number of patients in Analysis section and randomized number, there might be no missing data. | The outcome measure "Rate of patients of nitroglycerin suspension and reduction" in Method section were not reported in Analysis section. | It seemed to be true that article said "Baseline of participants was balanced." And others are probably suitable to low risk. |
| **ZHANG L 2011*** | The author told that Random number table was used to randomize the patients into 2 groups. | The author told that Opaque envelope was used to do the allocation concealment. | The author told there was no blinding on patients in the trials. | The author told there was no blinding on outcomes assessors in the trials. | Based on checking the number of patients in Analysis section and randomized number, there might be no missing data. | Based on checking the outcome measures in Analysis and Method sections, there might be no selective report. | Author said "Baseline of participants was balanced." And others are probably suitable to low risk. |
| **ZHANG LJ 2005*** | Unclear only based on "randomized into 3 groups"; author was not contacted. | No mention was in the paper; and the author was not contacted. | The trials compared acupuncture as adjuvant to medicine versus that medicine alone or compared acupuncture versus medicine. It was impossible to blind participants and acupuncturists. | No mention was in the paper; and the author was not contacted. | Based on checking the number of patients in Analysis section and randomized number, there might be no missing data. | Based on checking the outcome measures in Analysis and Method sections, there might be no selective report. | It seemed to be true that article said "Baseline of participants was balanced." And others are probably suitable to low risk. |

* Asterisk referred to the trials whose authors have been contacted by call for more information; # referred to that all the trials did not have the bias due to early stopping with benefits;

Green ball referred to the low risk of bias; Yellow ball referred to the unclear risk of bias; Red ball referred to the high risk of bias. The balls in “random sequence”, “allocation concealment”, blinding of participants”, “blinding of outcome assessment”, “incomplete outcome data”, “selective reporting” and other bias” referred to the risks of bias in the domains; and the balls in “Study ID” referred to the risk of bias for the trials.
