# Supplementary material for: Effectiveness of acupuncture for angina pectoris: a systematic review of randomized controlled trials
Source: BMC Complement Altern Med. 2015 Mar 28;15:90. doi: 10.1186/s12906-015-0586-7 (PMC4426772; doi:10.1186/s12906-015-0586-7)
Supplement: Additional file 3: — Effect estimates of acupuncture treatment in 25 included trials. [file 12906_2015_586_MOESM3_ESM.docx]

**Attachment 3** Effect estimates of acupuncture treatment in 25 included trials

| **Study ID** | **Comparisons** | **Outcomes time point (days)** | **Effect estimate (Random Effect, CI 95%)** | **P value** | **Heterogeneity** |
| --- | --- | --- | --- | --- | --- |
| ACUPUNCTURE VERSUS WESTERN MEDICINES | |  |  |  |  |
| **1 THE NUMBER OF PATIENTS SHOWING INEFFECTIVENESS OF ANGINA RELIEF** | | | | | |
| CHANG PF 2005 [43] | Body acupuncture versus western medicine | 14 | 0.55 [0.14, 2.21] |  |  |
| LIU YF 2012 [54] | Body acupuncture versus western medicine | 28 | 0.30 [0.09, 0.99] |  |  |
| **META-ANALYSIS** |  |  | RR 0.39 [0.16, 0.96] | P=0.04 | I^2^=0% |
| **2 THE NUMBER OF PATIENTS SHOWING NO ECG IMPROVEMENT** | | | | | |
| CHANG PF 2005 [43] | Body acupuncture versus western medicine | 14 | 1.10 [0.54, 2.23] |  |  |
| LIU YF 2012 [54] | Body acupuncture versus western medicine | 28 | 0.40 [0.18, 0.90] |  |  |
| **META-ANALYSIS** |  |  | RR 0.68 [0.25, 1.84] | P=0.44 | I^2^=71% |
| **3 NO. PATIENTS OF NTG SUSPENSION** | |  |  |  |  |
| CHANG PF 2005 [43] | Body acupuncture versus western medicine | 14 | RR 1.28 [0.43, 3.85] | P=0.66 |  |
| **4 NO. PATIENTS OF NTG REDUCTION** | |  |  |  |  |
| CHANG PF 2005 [43] | Body acupuncture versus western medicine | 14 | RR 1.24 [0.39, 3.98] | P=0.72 |  |
| ACUPUNCTURE VERSUS CHINESE MEDICINE | |  |  |  |  |
| **1 THE NUMBER OF PATIENTS SHOWING INEFFECTIVENESS OF ANGINA RELIEF** | | | | | |
| HUANG J 2004 [46] | Electroacupuncture versus Compound Danshen Pills | 28 | 0.71 [0.25, 2.06] |  |  |
| HUANG J2 2004 [47] | Electroacupuncture versus Compound Danshen Pills | 28 | 0.71 [0.27, 1.88] |  |  |
| LIU JR 2010 [51] | Body acupuncture versus Compound Danshen injection | 28 | 0.87 [0.42, 1.80] |  |  |
| WU CY 2009 [58] | Body acupuncture versus Compound Danshen Pills | 34 | 0.91 [0.41, 2.00] |  |  |
| ZHANG LJ 2005 [65] | Body acupuncture versus Compound Danshen Pills | 60 | 1.20 [0.40, 3.62] |  |  |
| **META-ANALYSIS** |  |  | RR 0.86 [0.58, 1.29] | P=0.47 | I^2^=0% |
| **2 THE NUMBER OF PATIENTS SHOWING NO ECG IMPROVEMENT** | | | | | |
| DIAO LH 2006 [44] | Electroacupuncture versus Compound Danshen Pills | 28 | 0.96 [0.51, 1.82] |  |  |
| HUANG J 2004 [46] | Electroacupuncture versus Compound Danshen Pills | 28 | 0.67 [0.34, 1.30] |  |  |
| HUANG J2 2004 [47] | Electroacupuncture versus Compound Danshen Pills | 28 | 0.89 [0.40, 1.98] |  |  |
| ZHANG LJ 2005 [65] | Body acupuncture versus Compound Danshen Pills | 60 | 1.11 [0.71, 1.72] |  |  |
| **META-ANALYSIS** |  |  | RR 0.94 [0.70, 1.27] | P=0.70 | I^2^=0% |
| **3 THE NUMBER OF PATIENTS WITH INEFFECTIVENESS OF DCG IMPROVEMENT** | | | | | |
| DIAO LH 2006 [44] | Electroacupuncture versus Compound Danshen Pills | 28 | 0.77 [0.40, 1.47] |  |  |
| WANG PJ 2011 [56] | Electroacupuncture versus Compound Danshen Pills | 28 | 0.60 [0.31, 1.15] |  |  |
| **META-ANALYSIS** |  |  | RR 0.68 [0.43, 1.08] | P=0.10 | I^2^=0% |
| **4 DCG MYOCARDIAL ISCHEMIA DURATION (mins)** | |  |  |  |  |
| WU CY 2009 [58] | Body acupuncture versus Compound Danshen Pills | 34 | MD -1.86 [-30.74, 27.02] | P=0.90 |  |
| **5 FRENQUENCY OF ANGINA ATTACKS (times/day)** | | | | | |
| HUANG J 2004 [46] | Electroacupuncture versus Compound Danshen Pills | 28 | MD -0.40 [-1.00, 0.20] | P=0.19 |  |
| **6** **NTG CONSUMPTION (tablets/day)** | | | | | |
| HUANG J 2004 [46] | Electroacupuncture versus Compound Danshen Pills | 28 | -0.40 [-0.90, 0.10] |  |  |
| LIU JR 2010 [51] | Body acupuncture versus Compound Danshen injection | 28 | -0.42 [-0.75, -0.09] |  |  |
| **META-ANALYSIS** |  |  | MD -0.41 [-0.69, -0.14] | P=0.003 | I^2^=0% |
| **7 NO. PATIENTS OF NTG SUSPENTION** | |  |  |  |  |
| WU CY 2009 [58] | Body acupuncture versus Compound Danshen Pills | 34 | RR 0.60 [0.15, 2.41] | P=0.47 |  |
| **8 NO. PATIENTS OF NTG REDUCTION** | |  |  |  |  |
| WU CY 2009 [58] | Body acupuncture versus Compound Danshen Pills | 34 | RR 0.89 [0.45, 1.75] | P=0.73 |  |
| ACUPUNCTURE PLUS OTHER INTERVENTIONS VERSUS OTHER INTERVENTIONS | | | | | |
| **1 ACUPUNCTURE PLUS WESTERN MEDICINES VERSUS WESTERN MEDICINES** | | | | | |
| **1.1 THE NUMBER OF PATIENTS SHOWING NO ECG IMPROVEMENT** | |  |  |  |  |
| CAO JP 2002 [42] | Body acupuncture plus western medicines versus western medicines | 15 | 0.70 [0.29, 1.70] |  |  |
| LI CP 2005 [48] | Body acupuncture plus western medicines versus western medicines | 28 | 0.47 [0.25, 0.90] |  |  |
| LIU JL 2007 [50] | Needle-embedding plus western medicines versus western medicines | 14 | 0.60 [0.25, 1.44] |  |  |
| LIU WP 2004 [52] | Body acupuncture plus western medicines versus western medicines | 28 | 0.27 [0.06, 1.19] |  |  |
| XU GD 2006 [61];  TONG YH1 2005 [62] | Electroacupuncture plus western medicines versus western medicines | 42 | 0.33 [0.15, 0.74] |  |  |
| YU W 2006 [63] | Electroacupuncture plus western medicines versus western medicines | 10 | 0.58 [0.27, 1.28] |  |  |
| ZHANG L 2011 [64] | Body acupuncture plus western medicines versus western medicines | 28 | 0.36 [0.14, 0.90] |  |  |
| **META-ANALYSIS** | |  | RR 0.47 [0.34, 0.65] | P<0.0001 | I^2^=0% |
| **1.2 THE NUMBER OF PATIENTS SHOWING INEFFECTIVENESS OF ANGINA RELIEF** | |  |  |  |  |
| CAO JP 2002 [42] | Body acupuncture plus western medicines versus western medicines | 15 | 0.35 [0.10, 1.24] |  |  |
| LI CP 2005 [48] | Body acupuncture plus western medicines versus western medicines | 28 | 0.36 [0.14, 0.91] |  |  |
| LIU JL 2007 [50] | Needle-embedding plus western medicines versus western medicines | 14 | 0.25 [0.06, 1.08] |  |  |
| LIU WP 2004 [52] | Body acupuncture plus western medicines versus western medicines | 28 | 0.38 [0.08, 1.79] |  |  |
| XU GD 2006 [61]; | Electroacupuncture plus western medicines versus western medicines | 42 | 0.19 [0.07, 0.56] |  |  |
| TONG YH1 2005 [62] |  |  |  |  |  |
| YU W 2006 [63] | Electroacupuncture plus western medicines versus western medicines | 10 | 0.55 [0.23, 1.28] |  |  |
| ZHANG L 2011 [64] | Body acupuncture plus western medicines versus western medicines | 28 | 0.32 [0.09, 1.10] |  |  |
| **META-ANALYSIS** | |  | RR 0.35 [0.23, 0.53] | P<0.00001 | I^2^=0% |
| **1.3 DCG MYOCARDIAL ISCHEMIA DURATION** | |  |  |  |  |
| CAO JP 2002 [42] | Body acupuncture plus western medicines versus western medicines | 15 | MD-16.94 [-49.64, 15.76] | P=0.31 |  |
| **1.4 FRENQUENCY OF ANGINA ATTACKS (times/week)** | | | | | |
| YU W 2006 [63] | Electroacupuncture plus western medicines versus western medicines | 10 | MD -0.43 [-6.90, -1.70] | P=0.001 |  |
| **1.5 DURATION OF ANGINA RELIEF (mins)** | | | | | |
| XU GD 2006 [61]; | Electroacupuncture plus western medicines versus western medicines | 42 | MD -1.40 [-1.62, -1.18] | P<0.00001 |  |
| TONG YH1 2005 [62] |  |  |  |  |  |
| **1.6 DURATION OF ANGINA DISAPPEARENCE (mins)** | | | | | |
| XU GD 2006 [61]; | Electroacupuncture plus western medicines versus western medicines | 42 | MD -5.30 [-6.53, -4.07] | P<0.00001 |  |
| TONG YH1 2005 [62] |  |  |  |  |  |
| **1.7 NO. PATIENTS OF NTG SUSPENSION** | |  |  |  |  |
| LIU JL 2007 [50] | Needle-embedding plus western medicines versus western medicines | 14 | RR 1.75 [1.06, 2.88] | P=0.03 |  |
| **1.8 NO. PATIENTS OF NTG REDUCTION** | |  |  |  |  |
| LIU JL 2007 [50] | Needle-embedding plus western medicines versus western medicines | 14 | RR 1.33 [1.04, 1.72] | P=0.03 |  |
| **1.9 EF (%)** |  |  |  |  |  |
| TONG YH 2005 [55] | Electroacupuncture plus western medicines versus western medicines | 42 | MD 1.04 [0.68, 1.40] | P<0.00001 |  |
| ZHANG L 2011 [64] | Body acupuncture plus western medicines versus western medicines | 28 | MD 1.89 [1.30, 2.49] | P<0.00001 |  |
| **1.10 QAULITY OF LIFE** | | | | | |
| **SYMPTOM** |  |  |  |  |  |
| LIU WP 2003 [53] | Body acupuncture plus western medicines versus western medicines | 28 | -3.90 [-5.64, -2.16] | P<0.00001 |  |
| XU GD 2006 [61]; | Electroacupuncture plus western medicines versus western medicines | 28 | -3.90 [-4.24, -3.56] | P<0.00001 |  |
| TONG YH1 2005 [62] |  |  |  |  |  |
| **META-ANALYSIS** |  |  | MD -3.90 [-4.23, -3.57] | P<0.00001 | I^2^=0% |
| **EMOTION** |  |  |  |  |  |
| LIU WP 2003 [53] | Body acupuncture plus western medicines versus western medicines | 28 | -1.90 [-5.18, 1.38] | P=0.26 |  |
| XU GD 2006 [61]; | Electroacupuncture plus western medicines versus western medicines | 28 | -1.90 [-3.06, -0.74] | P=0.001 |  |
| TONG YH1 2005 [62] |  |  |  |  |  |
| **META-ANALYSIS** |  |  | MD -1.90 [-2.99, -0.81] | P=0.0006 | I^2^=0% |
| **PERCEIVING ABILITY** | | | | | |
| LIU WP 2003 [53] | Body acupuncture plus western medicines versus western medicines | 28 | -0.40 [-2.29, 1.49] | P=0.68 |  |
| XU GD 2006 [61]; | Electroacupuncture plus western medicines versus western medicines | 28 | -0.40 [-0.82, 0.02] | P=0.06 |  |
| TONG YH1 2005 [62] |  |  |  |  |  |
| **META-ANALYSIS** |  |  | MD -0.40 [-0.81, 0.01] | P=0.06 | I^2^=0% |
| **SOCIAL STATUS** |  |  |  |  |  |
| LIU WP 2003 [53] | Body acupuncture plus western medicines versus western medicines | 28 | -0.50 [-2.18, 1.18] | P=0.56 |  |
| XU GD 2006 [61]; | Electroacupuncture plus western medicines versus western medicines | 28 | -0.20 [-0.49, 0.09] | P=0.18 |  |
| TONG YH1 2005 [62] |  |  |  |  |  |
| **META-ANALYSIS** |  |  | MD -0.21 [-0.49, 0.08] | P=0.15 | I^2^=0% |
| **BADL** |  |  |  |  |  |
| LIU WP 2003 [53] | Body acupuncture plus western medicines versus western medicines | 28 | 0.00 [-2.99, 2.99] | P=1.00 |  |
| XU GD 2006 [61]; | Electroacupuncture plus western medicines versus western medicines | 28 | 2.30 [1.27, 3.33] | P<0.0001 |  |
| TONG YH1 2005 [62] |  |  |  |  |  |
| **META-ANALYSIS** |  |  | MD 1.60 [-0.48, 3.67] | P=0.13 | I^2^=51% |
| **SENSE OF HEALTH AND HAPPINESS** | | | | | |
| LIU WP 2003 [53] | Body acupuncture plus western medicines versus western medicines | 28 | 14.40 [10.42, 18.38] | P<0.00001 |  |
| XU GD 2006 [61]; | Electroacupuncture plus western medicines versus western medicines | 28 | 14.40 [12.82, 15.98] | P<0.00001 |  |
| TONG YH1 2005 [62] |  |  |  |  |  |
| **META-ANALYSIS** |  |  | MD 14.40 [12.93, 15.87] | P<0.00001 | I^2^=0% |
| **WORK BEHAVIOR** | | | | | |
| LIU WP 2003 [53] | Body acupuncture plus western medicines versus western medicines | 28 | 7.00 [4.85, 9.15] | P<0.00001 |  |
| XU GD 2006 [61]; | Electroacupuncture plus western medicines versus western medicines | 28 | 7.00 [6.46, 7.54] | P<0.00001 |  |
| TONG YH1 2005 [62] |  |  |  |  |  |
| **META-ANALYSIS** |  |  | MD 7.00 [6.47, 7.53] | P<0.00001 | I^2^=0% |
| **SATISFACTION OF LIFE** | | | | | |
| LIU WP 2003 [53] | Body acupuncture plus western medicines versus western medicines | 28 | 3.10 [1.29, 4.91] | P=0.0008 |  |
| XU GD 2006 [61]; | Electroacupuncture plus western medicines versus western medicines | 28 | 6.10 [5.75, 6.45] | P<0.00001 |  |
| TONG YH1 2005 [62] |  |  |  |  |  |
| **META-ANALYSIS** |  |  | MD 4.74 [1.81, 7.66] | P=0.001 | I^2^=90% |
| **1.11 CARDIOVASCULAR EVENTS** | | | | | |
| XIE ZQ 2003 [60] | Electroacupuncture plus western medicines versus western medicines | 34 | RR 0.33 [0.16, 0.65] | P=0.001 |  |
| **2 ACUPUNCTURE PLUS CHINESE MEDICINE VERSUS CHINESE MEDICINE** | |  |  |  |  |
| **2.1 THE NUMBER OF PATIENTS SHOWING NO ECG IMPROVEMENT** | |  |  |  |  |
| DIAO LH 2006 [44] | Electroacupuncture plus Compound Danshen Pills versus Compound Danshen Pills | 28 | 0.25 [0.08, 0.81] |  |  |
| HUANG J2 2004 [47] | Electroacupuncture plus Compound Danshen Pills versus Compound Danshen Pills | 28 | 0.40 [0.13, 1.30] |  |  |
| LI HJ 2003 [49] | Body acupuncture plus compound Danshen injection versus compound Danshen injection | 28 | 0.63 [0.34, 1.18] |  |  |
| ZHANG LJ 2005 [65] | Electroacupuncture plus Compound Danshen Pills versus Compound Danshen Pills | 60 | 0.53 [0.28, 0.99] |  |  |
| **META-ANALYSIS** | |  | RR 0.51 [0.34, 0.75] | P=0.0006 | I2=0% |
| **2.2 THE NUMBER OF PATIENTS SHOWING INEFFECTIVENESS OF ANGINA RELIEF** | | | | | |
| HUANG J2 2004 [47] | Electroacupuncture plus Compound Danshen Pills versus Compound Danshen Pills | 28 | 0.43 [0.13, 1.43] |  |  |
| LI HJ 2003 [49] | Body acupuncture plus compound Danshen injection versus compound Danshen injection | 28 | 0.40 [0.08, 2.05] |  |  |
| ZHANG LJ 2005 [65] | Electroacupuncture plus Compound Danshen Pills versus Compound Danshen Pills | 60 | 0.20 [0.02, 1.64] |  |  |
| **SUBGROUP META-ANALYSIS** | |  | RR 0.37 [0.15, 0.89] | P=0.03 | I^2^=0% |
| **2.3 THE NUMBER OF PATIENTS WITH INEFFECTIVENESS OF DCG IMPROVEMENT** | |  |  |  |  |
| DIAO LH 2006 [44] | Electroacupuncture plus Compound Danshen Pills versus Compound Danshen Pills | 28 | 0.15 [0.04, 0.62] | P=0.009 |  |
| WANG PJ 2011 [56] | Electroacupuncture plus Compound Danshen Pills versus Compound Danshen Pills | 28 | 0.07 [0.01, 0.47] | P=0.007 |  |
| **META-ANALYSIS** |  |  | RR 0.12 [0.04, 0.36] | P=0.0002 | I^2^=0% |
| **2.4 NTG CONSUMPTION (tablets/day)** | | | | |  |
| LIU JR 2010 [51] | Body acupuncture versus Compound Danshen injection | 28 | MD-0.47 [-0.75, -0.19] | P=0.001 |  |
| **3 ACUPUNCTURE PLUS WESTERN AND CHINESE MEDICINE VERSUS WESTERN AND CHINESE MEDICINE** | | | | | |
| **3.1 THE NUMBER OF PATIENTS SHOWING NO ECG IMPROVEMENT** | |  |  |  |  |
| HU NK 1997 [45] | Electroacupuncture plus western and Chinese medicine versus western and Chinese medicine | 30 | 0.30 [0.10, 0.84] |  |  |
| WANG X 2000 [57] | Body acupuncture plus western and Chinese medicine versus western and Chinese medicine | 14 | 0.54 [0.20, 1.45] |  |  |
| WU HW 2005 [59] | Body acupuncture plus western and Chinese medicine versus western and Chinese medicine | 14 | 0.71 [0.39, 1.28] |  |  |
| **META-ANALYSIS** | |  | RR 0.56 [0.35, 0.90] | P=0.02 | I^2^=5% |
| **3.2 THE NUMBER OF PATIENTS SHOWING INEFFECTIVENESS OF ANGINA RELIEF** | | | | | |
| HU NK 1997 [45] | Electroacupuncture plus western and Chinese medicine versus western and Chinese medicine | 30 | 0.04 [0.00, 0.66] |  |  |
| WANG X 2000 [57] | Body acupuncture plus western and Chinese medicine versus western and Chinese medicine | 14 | 0.15 [0.02, 1.19] |  |  |
| WU HW 2005 [59] | Body acupuncture plus western and Chinese medicine versus western and Chinese medicine | 14 | 0.38 [0.11, 1.31] |  |  |
| **META-ANALYSIS** | |  | RR 0.20 [0.06, 0.69] | P=0.01 | I^2^=24% |
| **3.3 DURATION OF ANGINA RELIEF (mins)** | | | | | |
| WANG X 2000 [57] | Body acupuncture plus western and Chinese medicine versus western and Chinese medicine | 14 | MD -1.40 [-1.81, -0.99] | P<0.00001 |  |
| **3.4 DURATION OF ANGINA DISAPPEARENCE (mins)** | | | | | |
| WANG X 2000 [57] | Body acupuncture plus western and Chinese medicine versus western and Chinese medicine | 14 | MD -4.70 [-7.11, -2.29] | P=0.0001 |  |
| GENUINE ACUPUNCTURE VERSUS SHAM ACUPUNCTURE | | | | | |
| **1 GLOBAL EVALUATION SCALE AFTER TREATMENT** | | | | | |
| BALLEGAARD 1986 [35] | Genuine acupuncture versus sham acupuncture | 21 | RR 6.00 [0.85, 42.59] | P=0.07 |  |
| BALLEGAARD 1990 [36] | Genuine acupuncture versus sham acupuncture | 21 | RR 0.89 [0.67, 1.19] | P=0.44 |  |
| **2 GLOBAL EVALUATION SCALE AFTER 3 WEEKS FELLOW UP** | | | | | |
| BALLEGAARD 1990 [36] | Genuine acupuncture versus sham acupuncture | 21 | RR 0.93 [0.64, 1.35] | P=0.69 |  |
| **3 NTG CONSUMPTION** | | | | | |
| BALLEGAARD 1990 [36] | Genuine acupuncture versus sham acupuncture | 21 | RR 1.06 [0.89, 1.26] | P=0.51 |  |
| **4 ANGINA ATTACK RATE** | | | | | |
| BALLEGAARD 1990 [36] | Genuine acupuncture versus sham acupuncture | 21 | RR 0.99 [0.82, 1.20] | P=0.92 |  |
| **No. patients of Adverse events** | | | | | |
| HUANG J 2004 [46] | Electroacupuncture versus Compound Danshen Pills | 28 | RR 0.11 [0.01, 2.00] | P=0.92 |  |
