# Supplementary material for: Effectiveness of acupuncture for angina pectoris: a systematic review of randomized controlled trials
Source: BMC Complement Altern Med. 2015 Mar 28;15:90. doi: 10.1186/s12906-015-0586-7 (PMC4426772; doi:10.1186/s12906-015-0586-7)
Supplement: Additional file 5: — Meta-analyses of trials comparing acupuncture verse medicines in the no. patients with ineffectiveness of ECG. [file 12906_2015_586_MOESM5_ESM.docx]

**Attachment 5**

Meta-analyses of trials comparing acupuncture verse medicines in the no. patients with ineffectiveness of ECG
