# Supplementary material for: Effectiveness of acupuncture for angina pectoris: a systematic review of randomized controlled trials
Source: BMC Complement Altern Med. 2015 Mar 28;15:90. doi: 10.1186/s12906-015-0586-7 (PMC4426772; doi:10.1186/s12906-015-0586-7)
Supplement: Additional file 6: — Summary table of Sensitive analyses. [file 12906_2015_586_MOESM6_ESM.docx]

**Attachment 6 Summary table of Sensitive analyses**

| **Inclusion factors** | **Acupuncture plus others versus others** | | | | **Acupuncture versus medicines** | | | |
| --- | --- | --- | --- | --- | --- | --- | --- | --- |
|  | **the number of patients with ineffectiveness of ECG improvement (RR, 95%CI, numbers trials)** | | **the number of patients with ineffectiveness of symptom relief (RR, 95%CI, numbers trials)** | | **the number of patients with ineffectiveness of ECG improvement (RR, 95%CI, numbers of trials)** | | **the number of patients with ineffectiveness of symptom relief (RR, 95%CI, numbers trials)** | |
|  | **sensitive analysis** | **total meta analysis** | **sensitive analysis** | **total meta analysis** | **sensitive analysis** | **total meta analysis** | **sensitive analysis** | **total meta analysis** |
| Published trials | 0.49 [0.39, 0.63]  11 | 0.50 [0.40, 0.62]  14 | 0.27 [0.16, 0.46]  10 | 0.33 [0.23, 0.47]  13 | 0.85 [0.60, 1.21]  5 | 0.87 [0.65, 1.16]  6 | 0.77 [0.52, 1.14]  6 | 0.76 [0.53, 1.09]  7 |
| Sample size ≥ 40 | 0.49 [0.34, 0.72]  4 | 0.50 [0.40, 0.62]  14 | 0.22 [0.10, 0.45]  4 | 0.33 [0.23, 0.47]  13 | 0.91 [0.56, 1.49]  2 | 0.87 [0.65, 1.16]  6 | 0.91 [0.53, 1.58]  3 | 0.76 [0.53, 1.09]  7 |
| Explicit randomization procedure of contact information | 0.30 [0.16, 0.58]  3 | 0.50 [0.40, 0.62]  14 | 0.34 [0.13, 0.90]  2 | 0.33 [0.23, 0.47]  13 | 0.79 [0.44, 1.42]  3 | 0.87 [0.65, 1.16]  6 | 0.61 [0.31, 1.19]  3 | 0.76 [0.53, 1.09]  7 |
